# Advancements in the Preparation and Application of Ni-Co System (Alloys, Composites, and Coatings): A Review

**DOI:** 10.3390/nano15040312

**Published:** 2025-02-18

**Authors:** Liyan Lai, Feng Qian, Yuxiao Bi, Bing Niu, Guanliang Yu, Yigui Li, Guifu Ding

**Affiliations:** 1 School of Science, Shanghai Institute of Technology, Shanghai 201418, China; qianf1205@163.com (F.Q.); biyuxiao1999@163.com (Y.B.); bingniu1552@163.com (B.N.); glyu@sit.edu.cn (G.Y.); ygli@sit.edu.cn (Y.L.); 2 National Key Laboratory of Science and Technology on Micro/Nano Fabrication, School of Electronic Information and Electrical Engineering, Shanghai Jiao Tong University, Shanghai 200240, China

**Keywords:** non-silicon MEMS materials, nickel–cobalt composite material, electrodeposition, preparation method, mechanical/chemical properties

## Abstract

In the field of non-silicon MEMSs (micro-electro-mechanical systems), nickel, with its mature preparation method, good compatibility with non-silicon MEMS processes, and excellent mechanical properties, is one of the commonly used structural materials. By effectively combining it with non-silicon MEMS processes, nickel is widely used in typical process systems such as LIGA (Lithography, Galvanoformung, Abformung)/UV-LIGA (Ultraviolet Lithography, Galvanoformung, Abformung). However, with the rapid development of the non-silicon MEMS field, pure nickel materials are no longer able to meet current material demands. Alternatively, nickel–cobalt composite materials have excellent mechanical properties, thermal stability, corrosion resistance, and good adaptability to processing technology because cobalt has unique advantages as a reinforcing phase, including excellent wear resistance, corrosion resistance, and high hardness. This article examines the current methods for preparing nickel–cobalt alloys by focusing on composite electrodeposition of coatings and analyzing their advantages and disadvantages. Based on this, the effect of the composite electrodeposition conditions on the formation mechanism of nickel–cobalt alloy coatings is discussed. Then, the research status of composite electrodeposition methods mainly based on nickel–cobalt nanocomposites is discussed. Finally, a new direction for future work on nickel–cobalt composite materials mainly composed of nickel–cobalt nanomaterials prepared by composite electrodeposition is proposed, and its application prospects in non-silicon MEMS fields are discussed.

## 1. Introduction

Among many metallic materials, nickel (Ni) was initially widely used in the stainless-steel field and alloy steel because of its good corrosion resistance and high-temperature resistance. With the development of technology, the application areas of Ni are constantly expanding, for example, in the field of electroplating, by covering steel or other metal substrates with a layer of nickel plating, so as to improve its corrosion resistance; in the field of non-silicon MEMS materials, nickel (Ni) is one of the most commonly used materials in a variety of structural applications, primarily owing to the well-established and effective methods available for its electrodeposition, its efficient microfabrication process, its low-temperature process compatibility with the post-CMOS (complementary metal–oxide–semiconductor) process, and its excellent mechanical properties, making it a preferred material in numerous industrial applications. It has found extensive use in a range of typical process systems, such as LIGA/UV-LIGA, EFAB (electrochemical fabrication), and Metal-MUMPS (Metal Multi-User MEMS Processes) [1,2,3,4,5,6]. However, due to the rapid development of various industries, there are now higher standards for all aspects of material performance, and pure nickel materials are no longer capable of meeting the current material needs of many industries. Therefore, the nickel matrix composites prepared by adding metals [7,8,9,10], metal oxides [11,12,13], or non-metals [14,15,16,17] with excellent properties (such as high strength and hardness, corrosion resistance, and wear resistance, etc.) have been widely noticed and greatly developed.

Cobalt (Co) has excellent magnetic properties, wear resistance, and heat resistance, and is widely used in magnetic materials [18,19,20,21,22], cemented carbides [23,24,25,26,27], high-temperature alloys [28,29,30,31,32,33,34], battery materials [35,36,37,38,39], catalysts [40,41,42,43,44], and other fields. In terms of metal composites, Co, as an important component of the nickel–cobalt system, is usually added to the nickel matrix in the form of a solid solution to strengthen the matrix properties by changing the microstructure.

Based on the current research status, this article reviews the preparation methods of nickel–cobalt alloys, including nickel–cobalt nanoalloys. These methods include the sol–gel method, liquid-phase reduction, brush plating, mechanical alloying, and electrodeposition. Among these, electrodeposition has attracted wide attention. It is valued for its simple operation, controllability of electrocatalyst composition and morphology [45,46,47,48], and its ability to improve surface properties [49]. Additionally, electrodeposition causes less environmental pollution [50]. This paper investigates the influence of various processing parameters involved in the electrodeposition technique on the properties and characteristics of the resulting coatings, with a particular focus on nickel–cobalt nanocomposite materials.

## 2. Preparation of Nickel–Cobalt Alloys

As research into nickel–cobalt alloys continues to progress, the range of methods available for synthesizing these alloys has expanded significantly. The fabrication process of nickel–cobalt alloys is affected by numerous factors, including the selection of plating materials, variations in processing techniques, and the intricate chemical dynamics within the plating bath. Furthermore, the specific requirements for alloy precision, the limitations of the preparation environment, scalability for industrial production, and economic feasibility all play critical roles in determining the most suitable fabrication approach. Consequently, various methods are employed depending on the particular needs of the application, the available resources, and the properties desired of the final alloy. Currently, a number of well-established techniques are commonly used to produce nickel–cobalt alloys, each with its own advantages and challenges. These methods are tailored to achieve specific alloy characteristics, ranging from high corrosion resistance and wear durability to fine microstructural control for specialized uses. The following sections outline the most frequently adopted techniques for synthesizing nickel–cobalt alloys.

### 2.1. Sol–Gel Method

The sol–gel method [51] is implemented by first dissolving metal salts (e.g., metal alkoxides, nitrates, acetates, etc.) in a solvent, where the solvent can be an organic solvent, an aqueous solvent, or a mixture of organic and aqueous solvents coexisting together. The solvent is then allowed to gradually evaporate to form a sol and a gel; the dry gel is heat-treated to obtain the target product. In the initial stage of heat treatment, by heating at an appropriate temperature, organic solvents, alcohols, and water evaporate under heating, and residual organic components (such as alcohol groups, ether groups, etc.) decompose or evaporate. As the temperature gradually increases, further organic decomposition occurs, and metal precursors begin to transform into metal oxides, while their crystal structures also start to form. At higher temperatures, the crystallization of metal oxides is further improved, and the amorphous substances in the gel are transformed into a crystalline structure. The target product is usually a metal oxide, which is reduced to obtain a nickel–cobalt alloy.

Jiang Y et al. [52] utilized metal nitrates, citric acid, and ammonia as precursor materials to prepare dry gels through the conventional sol–gel technique, followed by activated self-combustion in a tube furnace at 300 °C to synthesize nickel–cobalt alloys. This approach combined both the sol–gel and self-combustion methods. The research highlighted the fuel-to-oxidizer ratio’s significant influence on the preparation process, showing that this ratio is a key factor in determining the outcome. Specifically, it was found that a fuel/oxidizer ratio of 1:1 yields the most favorable conditions for the successful formation of metals and metal alloys. This balance between fuel and oxidizer is critical for achieving optimal results, ensuring the desired chemical reactions occur efficiently and effectively.

Hua Z [53] synthesized a nickel–cobalt nanoalloy in a nitrate-based system, obtaining Co_0.5_Ni_0.5_. The particle size of the alloy ranged from tens to hundreds of nanometers, with a grain size of approximately 10 nm. A series of Co_1−x_Ni_x_ magnetic alloy powders were synthesized under a nitrogen atmosphere, and an appropriate amount of ethanol could increase the saturation magnetization strength to about 95% of the theoretical value. This experiment confirmed that the sol–gel technology can effectively prepare cobalt nickel magnetic alloys, and has the potential to synthesize other alloy systems.

The sol–gel method can obtain homogeneity at the molecular level in a very short time, and the reactants are capable of mixing uniformly at the molecular level as the gel forms. The product from the method is of high purity with a narrow particle size distribution and uniform nanostructures are realized at lower thermal conditions [54]. On the other hand, simultaneously, the prepared materials tend to be unstable and easily affected by chemical reactions or physical changes, such as the low efficiency of acid-catalyzed preparation and the disadvantages of inadequate material characteristics and the low structural strength of alkaline-catalyst-prepared surface coatings [55], in addition to the long reaction time, which can easily lead to uneven mixing of the substances and overgrowth of the crystals, which affects the quality and properties of the nanomaterials.

### 2.2. Liquid-Phase Reduction

The liquid-phase reduction approach involves dissolving the reactants in a solvent and introducing a reducing agent to the mixture of nickel–cobalt salts, while carefully regulating the reaction parameters. Through this reduction process, nickel–cobalt alloys can be synthesized by reducing the nickel–cobalt salts [56]. Commonly used reducing agents in this method include hydrazine hydrate, NaBH_4_, KBH_4_, and polyalcohols [56,57,58].

Usami T et al. [59] prepared a nickel–cobalt nanoalloy in a sulfate system containing hydrazine hydrate (N_2_H_4_-H_2_O) and trisodium citrate dihydrate (Na_3_C_6_H_5_O_7_-2H_2_O) and the phase structure was determined as Ni (FCC lattice structure), αCo (FCC crystal structure), and depended on variations in the Co:Ni ratio; at a Co–Ni molar ratio of 2:8, the lowest particle size of 50 nm was obtained.

He N et al. [60] successfully developed a series of cobalt–nickel alloy hollow microspheres composed of nanoparticles with adjustable composition and wall thickness using the liquid-phase reduction method. These microspheres had hexagonal HCP and FCC crystal structures. This study indicates that introducing nickel into a cobalt matrix induces a phase transition from HCP cobalt to FCC cobalt, significantly affecting various material properties such as particle size, internal strain, lattice parameters, alloy composition, and material thickness.

The advantages of the liquid-phase reduction method are the numerous sources of raw materials, ease of operation, and simple addition of the reductant to control the metal oxidation state without the need for high-temperature heat treatment [61]; but the disadvantages are the expensive price of the reductant sodium borohydride and the toxicity of hydrazine hydrate.

### 2.3. Brush Plating Method

The brush electroplating method is a plating process suitable for repairing or restoring damaged plating on high-strength steel components; it is an advanced process of surface technology [62]. The brush plating technology is simple to operate, and the growth mode of the coating shows intermittent crystallization, which leads to the generation of high-density dislocations, thus refining the grains; this process improves the nickel-based nanocomposite coating hardness and abrasion and corrosion resistance significantly.

Zheng Weiming et al. [63] used soluble-anode brush plating technology to produce nano-nickel–cobalt alloy coatings, combining electroplating with soluble anodes to ensure efficient deposition. The team conducted a series of characterizations, including X-ray diffraction (XRD), scanning electron microscopy (SEM), and energy dispersive spectroscopy (EDS), to analyze the structural and compositional characteristics of the coating. Their findings indicated that cobalt doping into the nickel matrix enhanced the mechanical and electrochemical properties of the coating. Specifically, adding Co promoted solid solution strengthening, resulting in finer grains within the coating structure. As the cobalt concentration in the alloy increased, the grain size continued to decrease, which in turn led to significant improvements in the microhardness, wear resistance, and corrosion resistance of the coating. The wear mechanism of the 36.14% Co-content nanocrystalline Ni-Co alloy coating was mainly adhesive wear. In comparison, the wear mechanism of the 44.16% Co-content nanocrystalline Ni-Co alloy coating exhibited abrasive wear characteristics. The grain size of Ni-Co alloy coatings prepared under the same brush plating process was in the nanometer range, and with an increase in Co content, the preferred orientation of the (111) texture was strengthened, and the grain size of the coating decreased while the hardness increased.

Fang X et al. [64] applied Ni-Co brush plating to the surface of stainless steel FV520(B) and employed scanning electron microscopy, mechanical testing, and nanoindentation techniques to assess the impact of an external magnetic field on the surface characteristics, alloy composition, tribological behavior, and residual stress. The findings indicated that the presence of a high-intensity magnetic field facilitated the formation of more uniform and defect-free nickel–cobalt alloy coatings. Moreover, as the magnetic field strength increased, the cobalt content in the alloy coating rose, while wear resistance improved and residual stress diminished. The optimal surface properties of the plated layer were achieved at a magnetic field strength of 0.2 T.

Brush plating technology comes with several advantages, including a fast plating speed, high plating quality, lightweight equipment, and simple process, and can be applied to on-site repair [65]. However, the disadvantages of this method are the complexity of the plating solution composition, the generally high cost, and the unsatisfactory performance of the plated layer [65,66].

### 2.4. Mechanical Alloying Method

Mechanical alloying is a way of preventing segregation and agglomeration of reinforcing materials and uniformly distributing the reinforcing particles into the metal alloy matrix [67,68,69]; a method to obtain a composite metal by mechanical force. It is used to mix the powder ingredients of pure metals, master alloys, and non-metals, the amounts of which are calculated according to the alloy composition. These ingredients are then processed in a dry high-energy ball mill. The grinding balls create strong collisions and churning effects. This results in the powders being repeatedly cold-welded and broken. The process creates composite metal powders with complex and uniform chemical compositions. It also results in fine microstructures. Mechanical alloying [70] was introduced as a method to produce a wide range of alloy phases, such as solid solutions, quasi-crystalline and crystalline intermetallic compounds, as well as amorphous structures.

Olvera S et al. [70] employed the high-energy ball milling technique to fabricate Co-Ni alloys, specifically Co_30_Ni_70_, Co_50_Ni_50_, and Co_70_Ni_30_, by blending cobalt (Co) and nickel (Ni) powders. This process involved the use of high-energy ball milling to induce alloy formation, followed by structural characterization. The findings revealed that the ball milling process induced vacancy formation, particularly around the cobalt atoms. These vacancies were believed to contribute to changes in the material’s atomic arrangement. Furthermore, a longer milling time decreased the crystalline size. The grain refinement observed during the milling process was attributed to the mechanical energy applied, which also enhanced the degree of ordering within the alloy. The study suggests that optimizing ball milling parameters could lead to improved properties for Co-Ni alloys in applications requiring fine microstructures and high material performance.

García-Contreras M A et al. [71] created a range of cobalt-nickel alloys with varying initial cobalt–nickel powder ratios. They conducted an extensive characterization of the resulting alloys using several analytical methods. Their findings revealed that after 5 h of ball milling, an FCC solid solution of cobalt and nickel began to form. After grinding for 20 h, they observed agglomerated particles with a size of about 10 nm. The study highlights mechanical alloying’s influence on the structural evolution of cobalt–nickel alloys, with the milling time making a substantial contribution to refining the grain size and enhancing the solid solution’s stable formation. The results suggest that control of milling times can be used to fine-tune the microstructure of cobalt–nickel alloys, which may have implications for their performance in various applications where small grain sizes and homogeneous compositions are desirable.

The primary benefit of the mechanical alloying method is its proficiency in generating large-scale solid materials (ranging from a few hundred milligrams to tons) with consistent physical properties, without the need for cooling from the liquid to the solid phase, thereby preventing the segregation of solutes [72]; but the disadvantage is that it is too costly when compared with methods such as the traditional heat treatment, and also mechanical alloying is affected by the material composition, the process parameters, and other factors, which have a limited impact on the material properties. In addition to this, the mechanical alloying process may produce pollutants such as exhaust gases [73], affecting the environment.

### 2.5. Composite Electrodeposition

Composite electrodeposition stands out as a crucial and cost-effective technique widely used in industry for the fabrication of protective coatings. Composite electrodeposition is a method of embedding small solid particles or nanomaterials into metal electroplating layers. It deposits metal on the surface of the substrate through electrochemical processes while uniformly embedding particulate materials (such as ceramic particles, carbon nanotubes, nanoparticles, etc.) in the electroplating layer, thereby improving the performance of the metal coating in terms of hardness, wear resistance, corrosion resistance, etc. The advantages of composite electrodeposition include its easy operation, simple equipment, low cost, high deposition rate, mild preparation conditions, uniform distribution of nickel and cobalt, and low amount of waste that is generated in the production process; in addition, this method is used in the field of MEMS applications, which can prepare a range of microstructures in the nanometer-to-millimeter range according to the size requirements, including single or multilayer structures, and monolayer/multilayer movable complex structures. Therefore, it is considered one of the essential approaches for fabricating metal matrix composites. Electroplated nickel and its alloys have found extensive applications across various industrial sectors [74,75,76,77].

**Figure 1:**
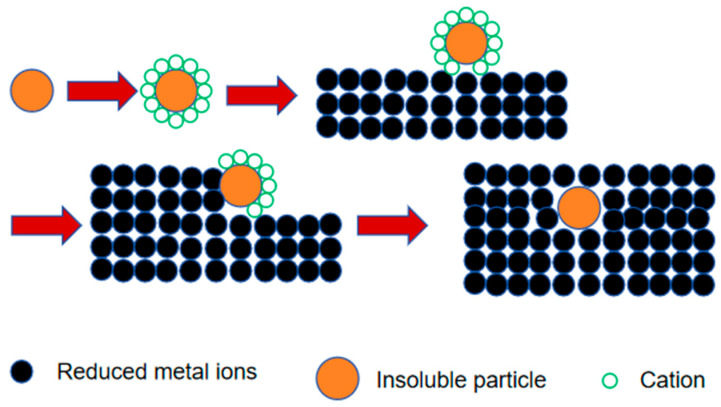
Adsorption process in tank-plated composite electrodeposition.

The current methods for realizing composite electrodeposition include slot-plating composite electrodeposition and nozzle jet composite electrodeposition [78]. As shown in Figure 1, the adsorption process in slot-plating composite electrodeposition follows Celis’ five-step adsorption principle: 1. The movement of charged cations adsorbed to the surface of insoluble particles, which makes the particles subsequently positively charged. 2. Free movement of the positively charged insoluble particle clusters in the plating solution. 3. Movement of the positively charged insoluble particle clusters through the boundary layer, which gradually spreads to the cathode. 4. Positively charged nanoparticle clusters are adsorbed by the cathode. 5. The adsorbed ions are reduced on the cathode surface, and the insoluble particle clusters are partially embedded into the plating layer to be completely deposited onto the composite plating layer. 6. The positively charged nanoparticle clusters are adsorbed by the cathode. The nozzle jet composite electrodeposition method uses the local high-speed controllable electrochemical processing technology, applying a high-speed jet of plating solution to the surface of the workpiece for electrodeposition; the impact of the plating solution on the deposited layer leads to effective activation; the formation of a dense, refined composite plating layer improves the efficiency of deposition by tens to hundreds of times [79].

The preparation methods of nickel–cobalt composites mainly used are the sol–gel method, liquid-phase reduction method, brush plating method, mechanical alloying method, and composite electrodeposition method. A comparison of these methods for preparing nickel–cobalt alloys is shown in Table 1. Among them, the composite electrodeposition method has been a commonly used preparation method in domestic and foreign research; using this method, the deposition rate (and thus the thickness), composition, and deposition characteristics of the composite layer can be well controlled [80,81,82,83], and the flatness of the composite layer obtained from the preparation of the surface is high. In addition, the composite electrodeposition method also has the advantages of a low preparation cost, stable coating structure, and less pollution. Therefore, the preparation of nickel–cobalt composites by the electrodeposition method has great potential, and the effects of process conditions on the plating layer and the current research status of this method in nickel–cobalt composites is discussed next.

## 3. The Effect of Process Conditions on Nickel–Cobalt Alloy Plating in Electrodeposition

### 3.1. The Effect of Cobalt Content on Nickel–Cobalt Alloy Plating Layer

In terms of the amount of cobalt present in the coating, Zhang Yicheng et al. [84] electrodeposited cobalt–nickel alloys using monophosphate and the study suggested an enhancement regarding the cobalt concentration in the electroplating bath of amino sulfonic acid and a decrease in cathodic current both led to a rise in the cobalt mass fraction in the plating layer, resulting in higher hardness and internal stresses in the layer. As the Co^2+^/(Ni^2+^ + Co^2+^) proportion of cobalt in the electrolyte solution increased, the Co concentration in the cobalt–nickel alloy plating was elevated. To safeguard the plating’s mechanical properties from stress-related deterioration due to high cobalt content, the Co concentration was limited to a maximum of 30%.

Wang Dan et al. [85] fabricated cobalt–nickel alloy coatings via pulsed electroplating using a sulfate system, the study suggested that with rising Co^2+^ concentration in the electroplating bath solution, the plating layer microstructure was transformed from an FCC structure to a mixed FCC and densely packed hexagonal structure (FCC + HCP); the hardness of the coating initially rose and subsequently declined as the cobalt content increased, with the optimal cobalt weight fraction being 59%. The concentration of cobalt in the Ni-Co coating rose with higher CoSO_4_ concentration in the electrolyte and with pulse frequency, while it decreased with increasing cathode current density.

**Figure 2:**
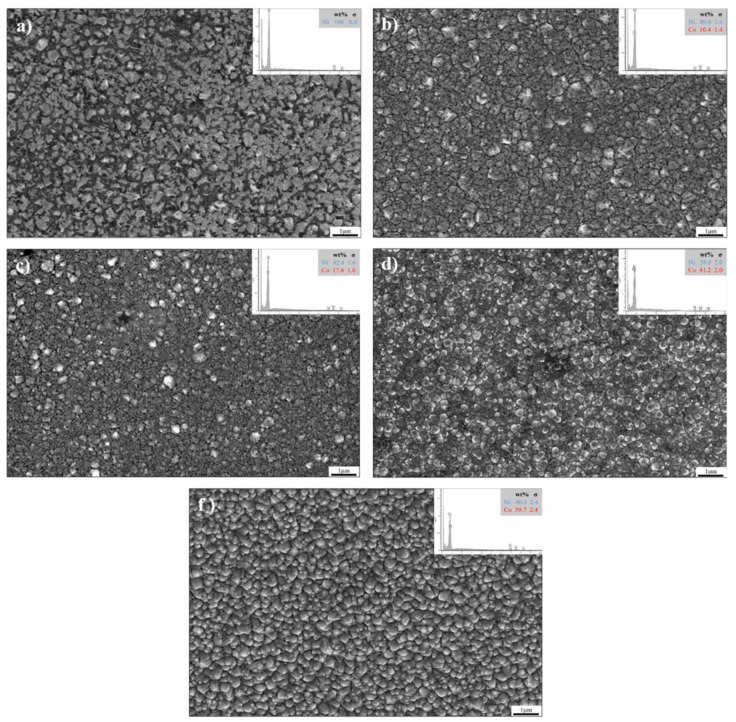
Surface morphology of nickel–cobalt alloy plating: (**a**) 0 g/L, (**b**) 10 g/L, (**c**) 20 g/L, (**d**) 60 g/L, and (**f**) 80 g/L addition [86].

Chen Y et al. [86] explored the fabrication of cobalt–nickel alloys by electrodeposition using a modified Watts electrolyte bath. The study primarily focused on alloys containing a cobalt content below 65 wt.%. The findings indicated that varying the cobalt concentration had minimal impact on the coating’s 3D nucleation and growth dynamics. Additionally, the cobalt content did not significantly alter the grain size. As Figure 2 shows, the grain size distribution at lower cobalt concentrations was irregular, with some larger grains present. As the cobalt content increased, the grain structure exhibited a more uniform distribution, and spherical particles predominated on the surface. As observed in Figure 2d, the coating’s surface exhibited a smoother appearance, characteristic of a more homogeneous particle distribution. However, further increases in cobalt content led to the formation of distinct protrusions with conical crystal structures on the coating surface, as shown in Figure 2f. Regarding the corrosion resistance of the cobalt–nickel alloys, the study found that the alloys demonstrated corrosion behavior similar to pure nickel, with the best corrosion performance observed at a cobalt concentration of 10 wt.%. Beyond this point, the cobalt content further increased, resulting in a noticeable decline in corrosion resistance. These findings suggest that while cobalt enhances certain properties of Ni-Co alloys, there is an optimal cobalt concentration for balancing mechanical and corrosion-resistant properties, after which the performance diminishes.

Ling W et al. [87] prepared cobalt–nickel alloys with varying cobalt content through pulsed electrodeposition. They fabricated four different cobalt–nickel alloys, each with a different cobalt concentration: 20 wt.% cobalt, 40 wt.% cobalt, 60 wt.% cobalt, and 80 wt.% cobalt. The concentration of Co^2+^ ions in the electrolyte solution was thoroughly examined to understand its influence on the cobalt content within the resulting electroplated layer. As illustrated in Table 2, the researchers observed a direct correlation: as the Co^2+^ ion concentration increased in the electrolyte, the cobalt concentration in the deposited layer also increased proportionally. This finding indicates that the cobalt content in the plated layer can be effectively controlled by adjusting the Co^2+^ concentration in the electroplating solution. Moreover, they evaluated the cobalt–nickel alloys’ electrocatalytic performance in various conditions. The outcomes demonstrated that the alloys exhibited optimal electrocatalytic activity when prepared under specific conditions, particularly at 10 mA/cm^2^.

To further examine the effect produced by fluctuations in Co content within the deposited layer on the performance of the coating, regarding the cobalt concentration in the plated layer, Yao Suwei et al. [88] prepared a nickel–cobalt alloy plating layer by electrodeposition through a sulfate system, and found that the alloy consisted of two solid solutions with FCC structure when the cobalt mass fraction of the plating layer was lower than 76%; when the cobalt mass fraction was between 76% and 90%, the alloy was composed of solid solutions that exhibited both FCC and HCP structures; when the cobalt mass fraction exceeded 90%, the nickel–cobalt alloy consisted of solid solutions with only one HCP structure. Wu Gang et al. [89] used electrodeposition of cobalt–nickel alloys with a sulfamic acid system electrolyte, and found that in the low-cobalt-content interval (Co < 25%), the structure of the cobalt–nickel alloy layer showed the cobalt dissolved in nickel formed by a single-phase solid solution that adopted an HCP structure; when the cobalt content was in the interval of 25–40%, the FCC structure of cobalt predominated; when the cobalt content surpassed 60%, the alloy layer primarily consisted of cobalt dissolved in nickel, forming an HCP structure. The structure of the alloy layer when the cobalt content was greater than 60% was a single-phase solid solution of cobalt with an HCP structure, formed by dissolving nickel in cobalt, which is basically consistent with the study of Yao Suwei et al. As the cobalt content surpassed 20%, the composition of the alloy underwent a marked transformation, leading to distinct modifications in its structural characteristics. At this level of cobalt concentration, the alloy exhibited a shift towards more stable phases, which significantly influenced its overall mechanical and physical properties; the coating’s hardness underwent a significant enhancement as the cobalt content was increased, reflecting a clear improvement in its mechanical strength; and when the proportion of cobalt present within the alloy composition was in the ranges of 20–50% and 60–80%, the FCC Co or HCP Co solid solutions had a higher value of hardness, and the cobalt–nickel alloy at this time could be used as a wear-resistant functional plating layer with a high degree of hardness.

**Figure 3:**
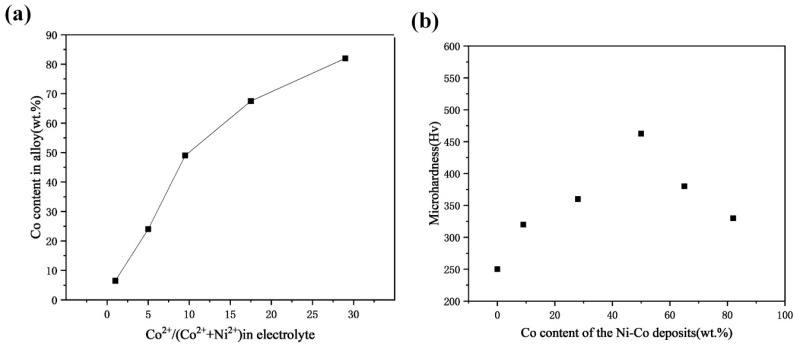
(**a**) Variation in alloy composition with Co^2+^ concentration in the plating solution. (**b**) Plated Co microhardness versus plated Co mass percentage [90].

Wang L et al. [90] also prepared the plating in a sulfate system by the electrodeposition method; it was found that the cobalt elemental proportion of the Ni-Co plating rose with the Co^2+^ concentration rise in the plating bath, as in Figure 3a, which is in agreement with the results of Ling W [87], and its microhardness, as in Figure 3b, showed a rise followed by a fall with an increase in the Co content of the plating, with the optimum concentration being 49 wt.%. It can be clearly observed that as the cobalt content increases from 7 wt.% to 49 wt.%, the Ni-Co alloy surface structure undergoes a noticeable transformation. Initially, the surface is characterized by uniform polyhedral microcrystals, which progressively evolve into spherical agglomerates as the cobalt concentration rises. Furthermore, when the cobalt content reaches 81 wt.%, the alloy surface morphology takes on a more intricate appearance, forming a well-defined branched structure. This gradual shift in surface features illustrates the significant influence that varying the cobalt level has on the alloy’s structural and morphological characteristics, reflecting a complex relationship between composition and material properties. The crystallographic arrangement of Ni-Co alloys undergoes a notable transformation as the cobalt content increases. Initially, the alloys exhibit an FCC phase structure, but as the proportion of cobalt rises, this structure gradually evolves into a hexagonal close-packed (HCP) arrangement. This phase transition is strongly influenced by the increasing cobalt concentration and is of great importance in determining the alloy’s mechanical properties and overall performance. Alloys with a higher cobalt content demonstrate a reduced friction coefficient and enhanced resistance to wear in comparison to those rich in nickel. This is attributed to the hexagonal crystalline structure present in the cobalt-rich alloys, which leads to a considerable decrease in friction and improved durability against wear.

**Figure 4:**
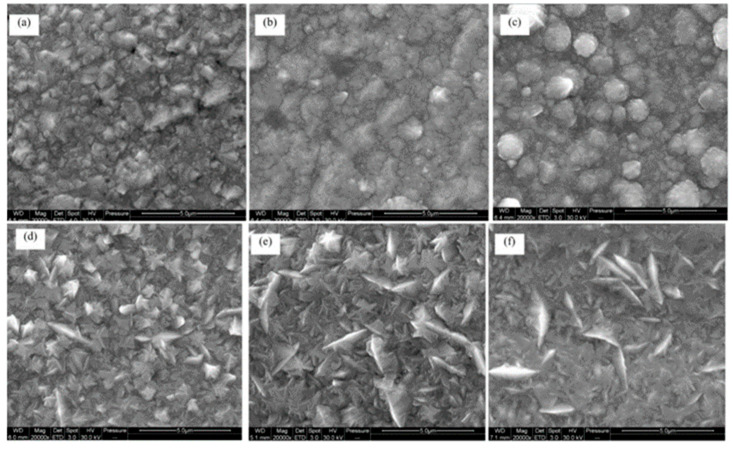
Surface morphology of coatings with different cobalt contents: (**a**) 2.5%; (**b**) 19.4%; (**c**) 39.6%; (**d**) 54.9%; (**e**) 71.9%; (**f**) 79.2% [91].

Wu Z W et al. [91], also using the sulfate system, obtained similar results. As shown in Figure 4, with the rise in cobalt content, the longitudinal cross-sectional morphology of the coating evolves from conical to spherical, and eventually to a poplar-like structure. The phase structure transitions from an FCC nickel solid solution to a face-centered cubic cobalt solid solution, and ultimately to an HCP cobalt solid solution. The microhardness of the alloy coating increases as the cobalt content rises, reaching a peak at a specific concentration, after which it begins to decrease. The maximum microhardness is observed at a cobalt concentration of 54.9%, marking the point of optimal hardness before the trend reverses with further increases in cobalt content. Additionally, the coating’s resistance to abrasion improves as the cobalt content increases.

Hong S H et al. [92] prepared a nickel–cobalt alloy on a copper substrate using the electrodeposition technique through a sulfate system and found that when the cobalt mass fraction in the plating was 15%, the structural size of this plating reached the minimum value; as the cobalt content in the cobalt–nickel alloy increased, a noticeable reduction was observed in the plating grain size. This trend continued up to a cobalt mass fraction of around 15%, where the grains became finer with higher cobalt concentrations. However, when the cobalt mass fraction exceeded 15%, the behavior changed. At this point, further increases in cobalt content led to the clustering of the grains, causing them to grow larger in size. This shift in grain structure suggests that the cobalt concentration significantly influences the nucleation and growth processes during the alloy electrodeposition.

Srivastava M et al. [93] studied the microstructural features and corrosion performance changes in an electrolytically deposited nickel coating during cobalt addition by electrodepositing cobalt–nickel alloys with varying cobalt concentrations using a sulfonate electrolyte. A cross-sectional microhardness analysis revealed that the hardness peaked at 50 wt.% cobalt concentration, which aligns with the grain size results for different Ni-Co alloys, as well as for coatings composed of pure nickel and cobalt, as presented in Table 3. It was found that the dimensions of the individual grains were smallest at 50 wt.% cobalt content. Optical images indicated that as the cobalt content increased, the microstructure evolved from a mixture of columnar fibers to a lamellar structure, and eventually to a fibrous arrangement; the analysis showed that the structure of the crystals remained cubic for cobalt concentrations between 0 and 50 wt.%. However, at cobalt contents of 70 wt.% and above, a transformation to a hexagonal arrangement was detected. Furthermore, the nickel–20% cobalt alloy exhibited superior corrosion resistance properties in this study compared to other nickel–cobalt alloys, as well as nickel and cobalt coatings with no impurities.

Zamani M et al. [94] investigated the synthesis of nickel–cobalt coatings with different cobalt concentrations through electrodeposition, using a modified Watts bath for the plating process. The data indicated that the amount of cobalt in the alloy coatings increased more quickly than in the plating bath. When the cobalt concentration reached 45% in the alloy, a significant reduction in grain size was observed, which led to improvements in both hardness and tensile strength. This grain refinement is generally associated with the strengthening of materials, as smaller grain sizes tend to inhibit dislocation movement, enhancing mechanical properties. However, as the cobalt content increased further to 55%, the coatings exhibited a slight decrease in both hardness and tensile strength, suggesting that an excess of cobalt can have detrimental effects on the alloy’s structural integrity. Among all the tested compositions, the Ni-25%Co alloy was particularly notable for its optimal properties, displaying a dense, tightly packed microstructure with smaller grain sizes. This dense structure contributed to its enhanced mechanical strength and durability. The study concluded that controlling the cobalt content in nickel–cobalt alloys is critical to achieving the desired balance between mechanical properties and microstructural characteristics, with the Ni-25%Co coating standing out as an ideal composition for applications requiring high material integrity and strength.

**Figure 5:**
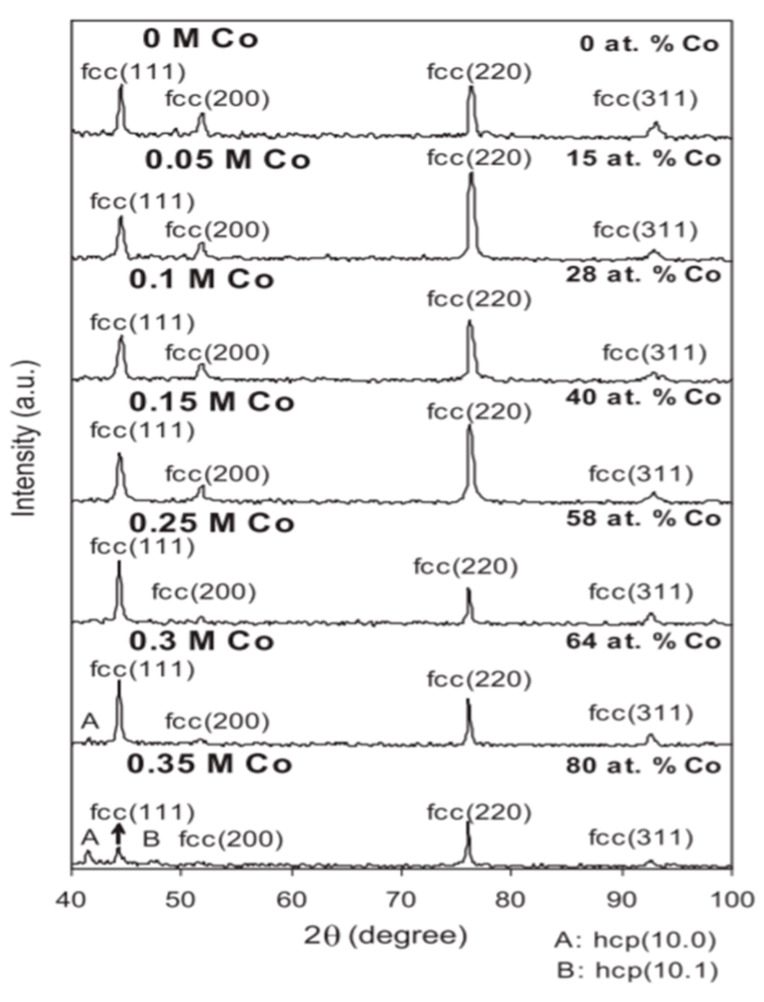
XRD patterns of Ni-Co thin coatings deposited by electrolytes with different Co concentrations [95].

Karpuz A et al. [95] explored the development of nickel–cobalt (Ni-Co) coatings through the electrodeposition process. The study aimed to investigate how variations in cobalt concentration within the electrolyte affected the resulting coatings’ physical and chemical properties. By systematically adjusting the cobalt content, the researchers were able to identify its impact on several key characteristics, including the microstructure, hardness, and overall performance of the deposited coatings. As the cobalt sulfate concentration increased, the cobalt content within the deposited coatings also rose proportionally. Figure 5 shows an XRD analysis showing that when the cobalt content ranged from 0 at. % to 58 at. %, the coatings exhibited a predominantly FCC crystal structure. However, at higher cobalt concentrations (64 at. % and 80 at. %), the coatings displayed a mixture of FCC and HCP phases, suggesting that the incorporation of cobalt led to phase transitions within the alloy structure. Changes in the crystal arrangement can have a notable effect on the mechanical and physical traits of the coatings. The surface morphology was also studied using SEM, which revealed that the coating’s roughness increased as the cobalt concentration was raised. This suggests that higher cobalt levels in the electrolyte not only influence the coating’s internal structure but also affect its surface texture, potentially altering its wear resistance and aesthetic appearance. Additionally, magnetic measurements indicated a gradual enhancement in the coating’s saturation magnetization as the cobalt content was increased, further underscoring the role of cobalt in enhancing the Ni-Co alloy coatings’ magnetic properties. This finding suggests that cobalt incorporation can be tailored to optimize a coating’s magnetic performance for applications that require specific magnetic characteristics. Based on the results, the researchers concluded that the optimal composition for Ni-Co coatings, in terms of both structural and magnetic properties, lies within the range of 28–40 at. % cobalt. Coatings within this composition range demonstrated a high magnetoresistance value, coupled with a smooth or only slightly granular surface morphology, making them perfect for applications that demand both high performance and excellent surface quality.

Tebbakh S et al. [96] prepared the electrodeposition of cobalt–nickel alloy coatings on ruthenium substrates, utilizing a chloride-based plating bath with a controlled pH of 3.8. Their primary goal was to explore how varying cobalt concentrations in the electrolyte affected the composition, structural characteristics, electrochemical properties, and overall microstructure of the deposited coatings. The study revealed that the cobalt content played a significant role in influencing the coatings’ properties, with noticeable changes in both their structure and performance as the cobalt concentration varied. As the cobalt concentration increased, alterations in the coatings’ surface morphology and crystallinity were observed. These changes were linked to the deposition parameters, including the composition of the electrolyte, which directly affected the formation of the alloy coating. The results showed that the coatings with higher cobalt content exhibited improved corrosion resistance compared to those with lower cobalt concentrations. This suggests that cobalt incorporation enhances the protective properties of the alloy, likely by influencing the microstructure and altering the coating’s passivation behavior. As the concentration of cobalt in the electrolyte was adjusted, significant shifts in the crystalline phases of the coatings were noted, with changes from one phase to another, such as from an FCC to an HCP structure, depending on the cobalt concentration. This observation highlights cobalt’s critical role in modulating the crystallographic phases of the cobalt–nickel alloy, which in turn influences the material’s mechanical, electrochemical, and thermal properties. Overall, the findings of this study underscore the importance of controlling the cobalt concentration in the electrodeposition process to achieve desired properties in cobalt–nickel alloy coatings. By adjusting the cobalt content in the plating bath, it is possible to fine-tune the microstructure, corrosion resistance, and electrochemical performance of coatings, making them suitable for various applications where these properties are critical, such as in electronics, energy storage devices, and corrosion-resistant coatings.

**Figure 6:**
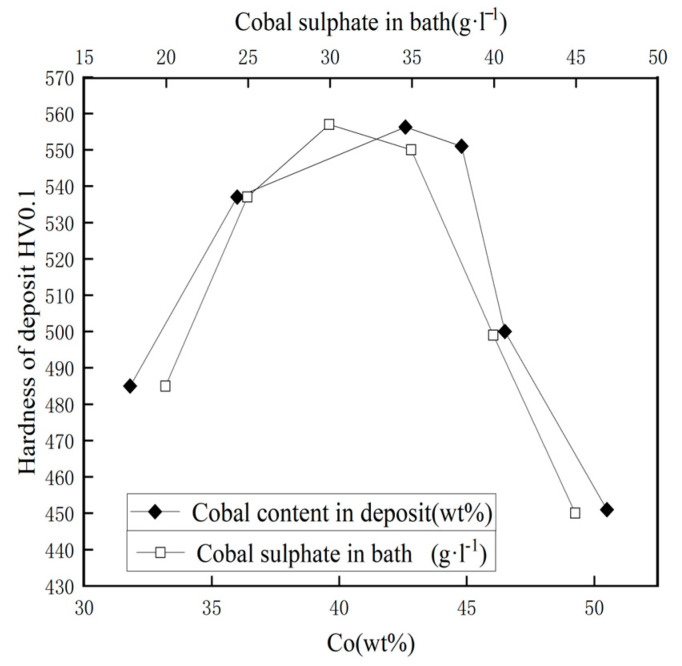
Relationship between the concentration of Co in the plating and sulfate bath and the HV0.1 hardness of the deposited plating [97].

Hagarova M et al. [97] investigated the electrodeposition of nickel–cobalt alloy coatings onto copper substrates from a sulfate bath, and their study revealed significant changes in the coatings’ properties as the cobalt sulfate concentration in the electrolyte was varied. As Figure 6 shows, they noted that the cobalt concentration in the coatings rose as the cobalt sulfate concentration in the solution was elevated, with the cobalt content rising from 31.8 wt.% to 49.2 wt.% when the concentration of CoSO_4_ in the electrolyte was adjusted from 20 g/L to 45 g/L. As the amount of cobalt in the electrolyte increased, there were distinct changes in both the texture and size of the grains. These alterations in the coating’s microstructure were strongly associated with the increasing cobalt content, suggesting that the cobalt concentration is a critical factor in controlling the morphological features and the overall grain structure of the alloy coatings. In particular, as the cobalt content increased, the coatings exhibited a finer grain structure, which is known to contribute to enhanced material properties such as hardness and wear resistance. Moreover, the electrodeposited nickel–cobalt alloy coatings’ hardness was directly influenced by the cobalt concentration in the plating solution. The interaction between the cobalt concentration in the sulfate bath and the coatings’ hardness, as measured by the Vickers hardness (HV0.1), was examined and showed that the highest hardness value of 556.3 HV0.1 was achieved at a cobalt content of approximately 42.6%, corresponding to a CoSO_4_ concentration of 30 g/L in the electrolyte. The results highlight the critical role of regulating the cobalt concentration throughout the electrodeposition process. By carefully adjusting this parameter, it becomes possible to optimize the nickel–cobalt alloy coatings’ mechanical characteristics and microstructure, thereby achieving the desired performance and durability. By adjusting the cobalt sulfate concentration in the electrolyte, it is possible to achieve coatings with the desired hardness and grain size, which is crucial for applications requiring high durability, such as wear-resistant coatings, electrical contacts, and corrosion-resistant materials.

The studies outlined above clearly demonstrate the significant influence of the cobalt concentration in the electroplating bath on the surface characteristics, structural integrity, and overall performance of the resulting electroplated coatings. However, the cobalt incorporation extent is not solely dependent on the cobalt content in the bath but is also influenced by factors such as the pulse frequency and cathodic current density. Higher pulse frequencies, for instance, tend to promote greater incorporation of cobalt into the plating, whereas increasing the cathodic current density tends to reduce the amount of cobalt in the deposited layer.

From a morphological standpoint, increasing the cobalt concentration in the plated layer leads to distinct changes in the surface structure. Initially, the longitudinal cross-section of the plating exhibits a conical shape. As the cobalt concentration rises, this shape evolves into a spherical form and eventually assumes a poplar-like morphology. In parallel with these changes in surface appearance, the crystallographic structure of the plating also undergoes a transformation. At lower cobalt concentrations, the coating predominantly exhibits an FCC crystal structure. As the cobalt content increases, however, a transition occurs, and the plating develops a dual-phase solid solution, incorporating both an FCC and a dense hexagonal close-packed (HCP) structure. When the cobalt content surpasses a certain threshold, the plating may even evolve into a fully hexagonal close-packed (HCP) structure, reflecting a significant alteration in the material’s crystallographic phase.

In terms of mechanical behavior, the hardness of electroplated coatings tends to increase as the cobalt content rises, primarily due to the solid-solution strengthening mechanism. Cobalt, with its larger atomic radius compared to nickel, creates lattice distortions when incorporated into the nickel matrix, thus enhancing the material’s overall hardness. This solid-solution strengthening becomes particularly prominent at intermediate cobalt concentrations, where the mechanical properties improve markedly. However, beyond a certain cobalt concentration, a decline in hardness is often observed. This stems from the weakening of the solid-solution phase as an excess of cobalt displaces nickel in the matrix, reducing the beneficial strengthening effect and causing the coating to lose its hardness.

The coating’s wear resistance follows a similar trend to hardness, initially improving with rising cobalt content but eventually deteriorating as the cobalt concentration increases beyond an optimal level. At lower cobalt concentrations, the wear mechanism is primarily governed by adhesive friction, while at higher concentrations, abrasive friction becomes the dominant factor. This shift in wear mechanism highlights the complex relationship between material composition and wear behavior. It is also important to note that the cobalt concentration that maximizes the hardness of the coating does not necessarily correspond to the highest wear resistance. The material wear resistance is influenced by a combination of factors such as hardness, plasticity, brittleness, and the overall surface structure.

For example, coatings with a dense, HCP structure tend to exhibit superior adhesion properties and a lower friction coefficient, leading to improved wear resistance. This underscores the multifaceted nature of material performance, where various structural and mechanical properties must be considered together. The interplay between the coating phase composition, coating microstructure, and coating mechanical properties, like coating hardness and wear resistance, makes it crucial to fine-tune the electroplating parameters, such as cobalt concentration, to achieve coatings with optimal performance in specific applications.

Ultimately, the research indicates that while increasing the cobalt content can enhance certain mechanical properties such as hardness and wear resistance, there exists an optimal cobalt concentration range where the properties are balanced most effectively. Beyond this point, excessive cobalt incorporation may have detrimental effects on the coating’s overall performance. Therefore, careful control over the electroplating process is essential to achieve coatings with the desired combination of structural integrity, mechanical strength, and wear resistance, particularly for applications in demanding environments where both durability and performance are critical.

### 3.2. Type of Current and Density Magnitude

Zhang Yicheng et al. [84] electrodeposited Ni-Co alloys using an aluminophosphate electrolyte system with a current density range of 2–10 A/dm^2^. The study revealed that as the cathodic current density increased, the cobalt concentration in the plated layer decreased. This behavior is likely due to the higher polarization of cobalt during the electrodeposition process than nickel. Because cobalt experiences a greater degree of polarization, its reduction at the cathode becomes more difficult at higher current densities, thereby reducing its incorporation into the plating. In contrast, nickel, which is less susceptible to polarization, is more readily deposited under these conditions, resulting in a higher nickel content in the plating. In their studies on Ni-Co alloy plating baths containing NiCl_2_ and CoCl_2_ as the primary salts, B. Tury et al. [98,99] observed that the pulse parameters significantly influenced the structure of the plated layer. They discovered that fine grains, greater compactness, and improved corrosion resistance could be achieved under conditions of lower current density and longer pulse-off times. Yanrong Chen et al. [100] investigated the pulse plating process for Ni-Co alloys using an electrolyte system based on NiSO_4_·6H_2_O and CoSO_4_·7H_2_O as the main salts. The researchers observed that the internal stress within the plated layer diminished as the duty cycle increased. This suggests that a higher duty cycle, which typically involves longer periods of current “on” time relative to “off” time, promotes a more stable deposition process, leading to a reduction in stress accumulation within the coating. On the other hand, they found that internal stress increased as the peak current density was raised. This can be attributed to the more intense conditions during deposition at higher current densities, which may lead to faster plating rates and greater atomic displacement, resulting in higher internal stress within the coating.

From this perspective, Liu Xuewu et al. [101] fabricated cobalt–nickel alloy coatings through electrodeposition using a high-frequency pulsed power supply, operating within a current density interval between 2 and 7 A/dm^2^. Their investigation aimed to evaluate how high-frequency-pulse plating compares to direct current (DC) plating in terms of coating characteristics. The study revealed that the coatings deposited via high-frequency pulsed plating were smoother, finer, and exhibited lower porosity compared to those produced by DC plating. These pulse-plated layers also demonstrated significantly improved abrasion resistance, with wear performance increasing further as the pulse frequency was raised. This enhancement can be attributed to the high-frequency pulses’ effects on grain growth inhibition and nucleation rate improvement. Higher pulse frequencies promote finer grain structures by increasing the number of nucleation sites, which reduces the porosity and enhances the overall densification of the coating, ultimately improving its resistance to wear.

Moreover, the study found that, under constant conditions, the average current density increase encouraged greater cobalt precipitation. Due to the short “off” time in high-frequency pulsed plating, the Co^2+^ concentration in the pulsed diffusion layer drops significantly during the on-cycle. The slow recovery of the Co^2+^ concentration during the off-time makes it challenging for the cobalt content to reach its original level in the solution, leading to the cobalt concentration reduction within the alloy layer as the average current density increases. The results indicate that while a higher current density can improve some aspects of deposition, it also influences the cobalt content of the alloy coating, which may affect both the final coating microstructure and performance.

Chen Y et al. [102] developed nanostructured cobalt–nickel alloy coatings using jet electrodeposition, aiming to investigate the effects of various processing parameters on the coating’s properties applied to 304 stainless-steel substrates. Their study found that current density played a dominant role in influencing the coatings’ deposition rates, microhardness, and surface roughness, with deposition time and scanning speed contributing to a lesser extent. Through careful experimentation and evaluation of these factors, the researchers were able to determine the optimal process parameters for achieving coatings with desirable properties: a deposition time of 20 min, a current density of 70 A/dm^2^, and a scanning speed of 10 mm/s. Under these optimal conditions, the resulting cobalt–nickel alloy coating exhibited a microhardness value of 532.40 HV, indicative of its superior mechanical strength. Additionally, the coating demonstrated an impressively low surface roughness of just 85 nm, reflecting a smooth and uniform finish. The adhesion strength of the coating was also measured at 26.12 N, showcasing its strong adherence to the stainless-steel substrate. This suggests that the coating could withstand mechanical stress and physical wear without delaminating, making it highly durable for practical applications. As far as corrosion resistance is concerned, the cobalt–nickel coating performed exceptionally well. The self-corrosion current density was found to be 5.718 × 10^−6^ A/cm^2^, signifying the coating’s excellent ability to resist corrosion in aggressive environments. This is a crucial property, especially in industrial applications where materials are subjected to harsh chemical conditions and corrosion over time. Overall, the study underscores the importance of carefully optimizing the deposition parameters to achieve coatings with outstanding mechanical properties, low surface roughness, strong adhesion, and excellent corrosion resistance. These findings highlight how the jet electrodeposition technique can be tailored to produce high-performance Ni-Co alloy coatings, with the potential for a range of industrial applications that demand durability and long-term reliability. By controlling process variables like current density, deposition time, and scanning speed, it is possible to enhance the quality of the coatings and tailor them to specific performance requirements.

Li Y et al. [103] explored the electroplating of nano-Ni-Co coatings from solutions with saccharin and cobalt sulfate as components under fixed electrodeposition parameters (pulse duration of 1 ms for on-time and 15 ms for off-time). The study revealed that increasing the peak current density led to a reduction in the deposited cobalt content, along with a more distinct colony-like surface morphology, smaller grain sizes, and enhanced hardness and tensile strength. These improvements were attributed to the higher overpotential and increased nucleation rate resulting from the elevated peak current density. However, further increases in peak current density were found to decrease both the hardness and tensile strength. Nanostructured cobalt–nickel alloy coating grain sizes between 15 and 20 nm could be produced using peak current densities ranging from 100 to 120 A/dm^2^, yielding hardness values between 590 and 600 kg/mm^2^ and tensile strengths between 1180 and 1200 MPa. These values were considerably higher than those of coatings made from pure nickel alloy prepared under similar conditions, despite having comparable grain sizes.

In experimental studies controlling the current density, the coating’s surface morphology can be significantly altered, leading to smoother and more refined finishes. An increase in current density not only refines the surface structure but also enhances metal dispersion across the coating. This helps to reduce porosity, which in turn contributes to the mechanical robustness of the coating, improving its toughness. As a result, higher current densities generally lead to an increase in the coating hardness and coating wear resistance of the plated layer, making the coating more durable and suited for demanding applications.

Moreover, optimizing the current density also helps achieve a more uniform alloy composition, which improves the adhesion between the coating and the substrate. This uniformity ensures better interfacial bonding, reducing the likelihood of delamination or poor adhesion under mechanical stress. Therefore, a well-controlled current density is essential for maintaining the cobalt–nickel alloy coating’s integrity and performance. However, there are limits to the benefits of increasing current density. When the current density exceeds a threshold value, typically around 2 A/dm^2^, the cobalt content in the plated layer starts to decrease. This reduction in cobalt concentration negatively impacts the coating’s properties. As the cobalt content decreases, the coating’s mechanical and electrochemical performance deteriorate, which may lead to a decrease in hardness, wear resistance, and corrosion resistance. Therefore, while higher current densities initially improve the coatings’ deposition quality and performance, excessive current densities can lead to undesirable effects, such as a reduction in the desired cobalt concentration and a decline in overall performance.

In conclusion, the current density is a critical parameter in the electrodeposition of cobalt–nickel alloy coatings. Achieving the optimal current density is crucial for obtaining coatings with the best combination of structural integrity, coating hardness, coating corrosion resistance, and coating wear resistance. The fine balance between improving dispersion, controlling composition, and maintaining the cobalt content within a desirable range is essential for producing high-performance coatings suitable for various industrial applications.

### 3.3. Other Process Parameters

Liu G et al. [104] dynamically supplemented Mn^2+^ ions into a lithium-ion battery plating liquid system while synergistically adjusting the pH in order to rapidly electrodeposit nickel–cobalt alloy coatings on the cathode. It was found that real-time pH control could effectively inhibit the negative effects of Mn^2+^-induced oxidation by pH reduction. In addition, the pH adjustment helped stabilize the cathode’s surface potential, which in turn improved its ability to attract Ni^2+^ and Co^2+^ ions. In a separate study, Tian L et al. [105] investigated the influence of pH and Co^2+^ concentration on the electrodeposition of nickel–cobalt alloy coatings on copper substrates. Their results indicated that as the pH increased within the range of 2.0 to 5.4, the current efficiency improved significantly, starting at 52.1% and going up to 81.2%. Simultaneously, the cobalt content in the deposited layer increased from 9.4% to 19.6%. Vazquez -ArenasJ et al. [106] investigated the electrolyte composition and pH effect on the co-deposition of Co in a sulfate solution using linear scanning voltammetry and cathodic polarization experiments.

Liang Yan et al. [107] used high-frequency pulsed current to prepare nickel–cobalt alloys. In terms of pH, it was found that maintaining a low pH within the permissible range results in a wide current density range, which can be increased accordingly by increasing the main salt content and increasing the temperature thereby employing higher current densities; this is favorable for anodic dissolution, and there are fewer pinholes in the plated layer.

**Figure 7:**
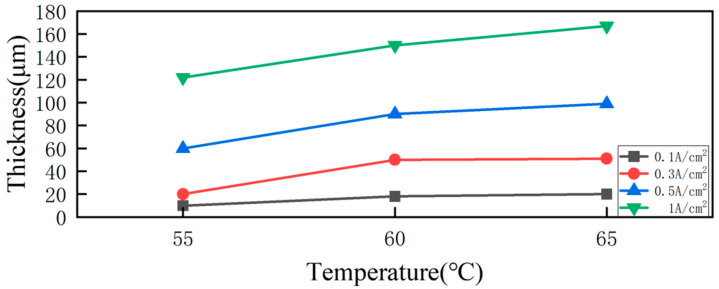
Temperature versus plating thickness [108].

Idris J et al. [108] investigated nickel–cobalt alloy coating electroplating in a Watts-type solution system, focusing on how temperature influences the deposited layer’s properties. The results showed that the thickness of the plated layer increased with higher plating temperatures, as depicted in Figure 7. This is due to the fact that elevated temperatures promote grain growth within the coating. Higher temperatures enhance the mobility of ions and atoms, which encourages larger grain formation and consequently leads to a thicker plating layer. Thus, the observed increase in the layer thickness is due to the temperature-driven enhancement of the grain structure during the electrodeposition process.

**Figure 8:**
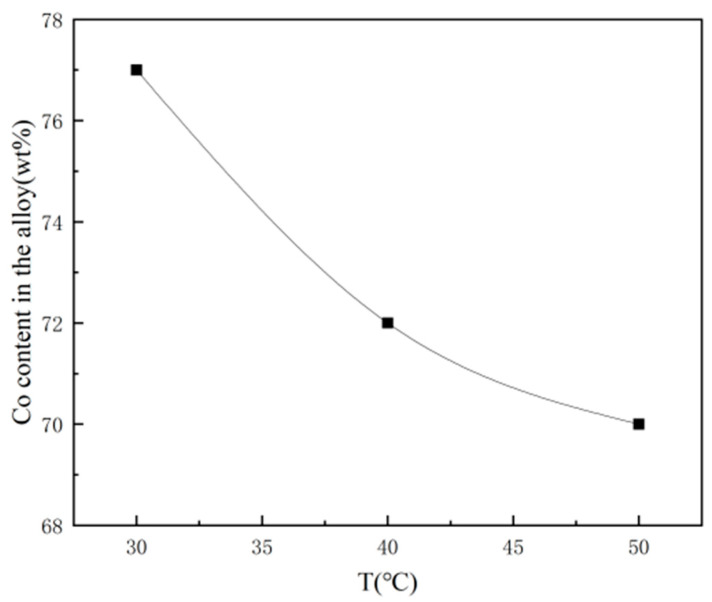
Effect of plating solution temperature on the composition of cobalt–nickel alloys [109].

**Figure 9:**
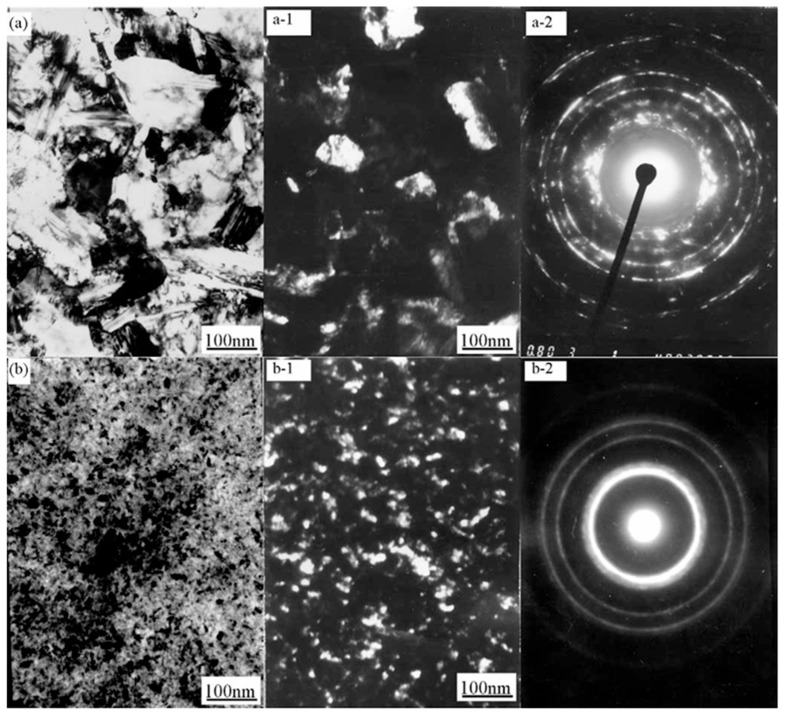
TEM micrographs and diffraction patterns for deposited Co-Ni alloys: (**a**) 159 A/dm^2^ and (**b**) 477 A/dm^2^ [109].

Qiao G et al. [109] developed nanocrystalline cobalt–nickel alloys through jet electrodeposition using an electrolyte jet device in a Watt’s bath, examining how the electrolyte temperature and jetting velocity influenced the cobalt–nickel alloys’ chemical composition and the structure of the Ni-Co alloy. As illustrated in Figure 8, their results showed that the deposition rate increased with rising bath temperature, and the cobalt content in the plated layer decreased with higher temperatures. This trend corroborates the findings of Idris J. They observed that increasing the electrolyte jetting speed led to a higher cobalt concentration in the deposited layer. This effect is due to higher jetting speeds enhancing the agitation of the electrolyte. The intensified stirring action reduces the diffusion layer thickness, leading to a higher metal ion concentration at the cathode, which in turn promotes the deposition of more cobalt in the alloy. Figure 9 shows plan view images of two samples deposited by applying different current densities of 159 and 477 A/dm^2^. The transmission electron microscope (TEM) images demonstrate that a nanostructured alloy was obtained. The average particle size of the image in Figure 9a is about 100 nm. In sharp contrast, the grain size in Figure 9b is approximately 10 nm. Obviously, with higher current density, the grain size of the deposited alloy decreases sharply.

The influence of different factors on the experimental results is shown in Table 4. Overall, the cobalt content and current density have a significant impact on the thickness, hardness, and uniformity of the coating. The pH value and temperature are important factors in regulating the composition and quality of the coating during the electroplating process, especially for obtaining a uniform and high-quality coating, appropriate pH value and temperature are crucial. The selection of current density type can further improve the microstructure and properties of the layer, and pulsed current usually brings better performance.

## 4. Research Status of Composite Electrodeposition Method in Nickel–Cobalt Composites

With the development of technology, nickel–cobalt alloys may not fully meet certain application requirements, such as hardness, wear resistance, corrosion resistance, etc. Based on nickel–cobalt alloys, introducing second-phase substances, especially nanoparticles or other reinforcing phases (such as carbon nanotubes, ceramic particles, metal nanoparticles, etc.), can endow nickel–cobalt composite coatings with characteristics different from electroplated alloys. Especially in nanomaterials, the nanoscale structure of nickel–cobalt nanocomposites results in higher strength, hardness, and wear resistance than conventional nickel–cobalt composites. Nanoscale particles can effectively prevent material deformation under stress, increasing tensile and compressive strength. The electronic and thermal conductivity of nanostructured materials are usually superior to macroscopic materials; therefore, nickel–cobalt nanocomposites may exhibit better performance in electronic devices and thermal management systems. The high surface energy and fine structure of nanocomposites may help improve their corrosion resistance, especially in acidic or alkaline environments, extending the service life of the material.

During the fabrication of nickel–cobalt compound materials, the composite electrodeposition method uses the electroplating method to deposit solid particles alongside the metal so as to obtain a composite plating layer with a diffusely distributed particle structure on the matrix metal on the substrate. In the electroplating process, solid, undissolved particles are uniformly dispersed throughout the plating bath, resulting in a stable suspension. This ensures that the suspended particles are evenly distributed within the solution, which is vital in facilitating the consistent deposition of metal ions onto the substrate, thereby promoting the formation of a uniform and high-quality electroplated layer. The composite electrodeposition technique is characterized by simplicity and convenience compared to the plating preparation methods like the sol–gel method, liquid-phase reduction method, brush plating method, and mechanical alloying method [80,110]. Whether the second-phase substances can be successfully compounded into the grown plating layer depends on many factors such as the characteristics of the substances themselves, the electrodeposition solution parameters, and so on [111,112,113,114,115]. Next, research on the preparation of nickel–cobalt composite materials is introduced, mainly focusing on the prepared nanomaterials and classified according to the introduced reinforcing phases.

### 4.1. Metals or Metallic Compounds

**Figure 10:**
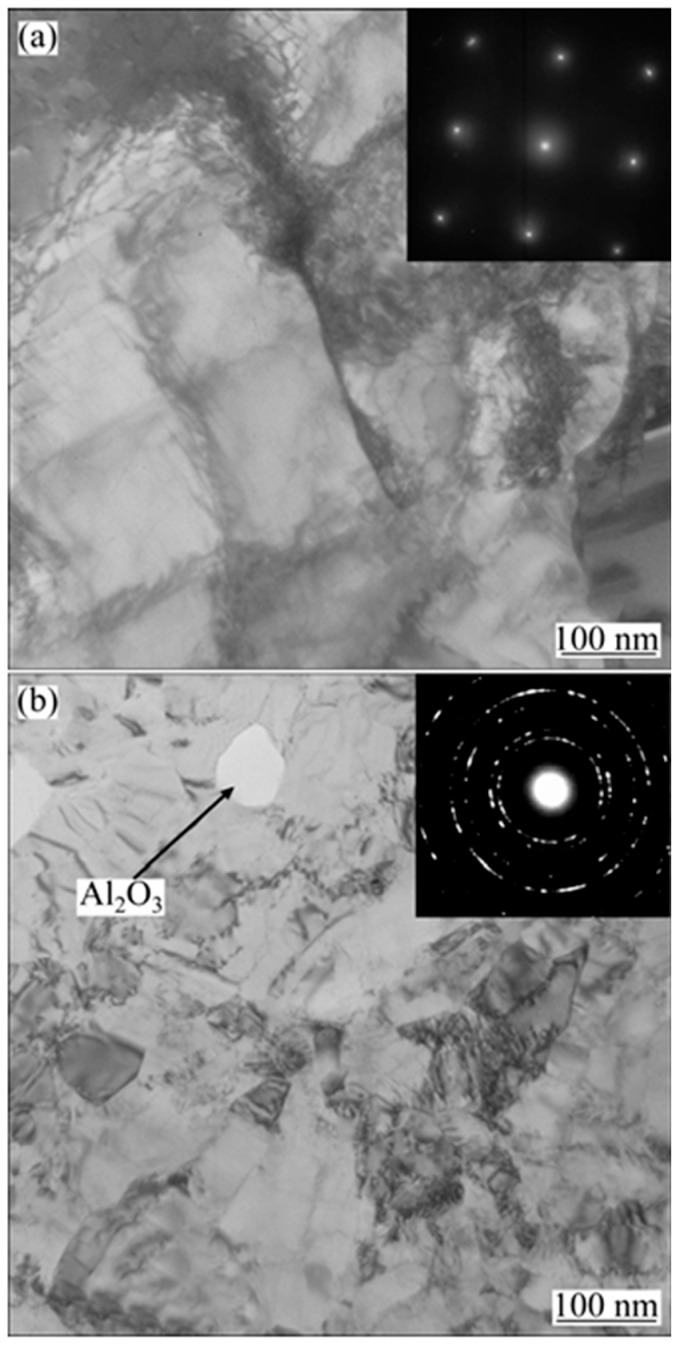
Bright-field TEM images showing deposited microstructures with different additives: (**a**) 1,4-butanediol; (**b**) saccharin [116].

WAN G G et al. [116] employed pulsed electrodeposition to fabricate Al_2_O_3_/Ni-Co nanocomposite materials, discovering that the nanoparticles’ reinforcing effect enhanced the composite’s microhardness. As the size of the Al_2_O_3_ particles increased, the yield strength slightly decreased, while strain hardening increased, which was attributed to the larger grain size. This suggests that with an increase in grain size, the material’s structural integrity weakens, making it more susceptible to slip, which in turn reduces the yield strength. In this study, the maximum tensile strength of 150 MPa was achieved when the grain size was reduced to 50 nm. Figure 10 shows TEM images of electrodeposited Al_2_O_3_/Ni-Co nanocomposites containing various additives. It is evident that compared to 1,4-butanediol, saccharin is more effective in producing finer particles. The average particle sizes of the deposited materials containing the 1,4-butanediol and saccharin additives are 180 nm and 37 nm, respectively. The selected diffraction pattern shown in Figure 10 is circular, confirming the ultrafine grain structure. In addition, circular gaps can be observed in Figure 10. This hole may have been formed due to the loss of Al_2_O_3_ particles during the sample preparation process.

Yang Tian et al. [117] used a composite electrodeposition method to incorporate chromium elements into Ni-Co alloy coatings. The investigation revealed that Cr^3+^, Ni^2+^, and Co^2+^ ions collaborate to enhance discharge and facilitate the co-deposition process, resulting in the formed nickel–cobalt–Cr alloy coatings exhibiting excellent high-temperature mechanical performance, improved oxidation resistance, and enhanced corrosion resistance. These coatings are capable of fulfilling the requirements for sustained and efficient operation under elevated temperature and pressure conditions.

Zhen Zhang et al. [118] fabricated a Ni-Co/Al composite coating through electrochemical co-deposition, and the influence of the Al particle concentration on the co-electrodeposition process and performance of the coatings was investigated. The resulting coatings exhibited dense and smooth surfaces with a nodule-like morphology composed of grain clusters. The grain size of the coatings was reduced from 30 nm to 25 nm with the addition of Al particles but increased from 27 nm to 38 nm as the current density was raised from 2 to 8 A/dm^2^. The average Ra values for the surfaces prepared at 2 A/dm^2^ and 6 A/dm^2^ were 191 nm and 302 nm, respectively, suggesting that higher current densities led to a rougher surface.

Li B et al. [119] employed a single-step DC electrodeposition method to fabricate nickel–cobalt and nickel–cobalt/ZrO_2_ composite coatings for use as protective layers. The results showed that the addition of ZrO_2_ to the Ni-Co matrix significantly enhanced both corrosion resistance and mechanical performance. Moreover, increasing the ZrO_2_ concentration led to a rise in the content of Co and ZrO_2_ within the coating. The study also found that a plating duration of less than 60 min was beneficial for reducing the nickel–cobalt/ZrO_2_ coating’s surface roughness. The hardness of the resulting nickel–cobalt alloys was notably affected by the current density, plating time, and ZrO_2_ content, with the inclusion of ZrO_2_ leading to a substantial increase in hardness compared to pure nickel–cobalt alloys.

**Figure 11:**
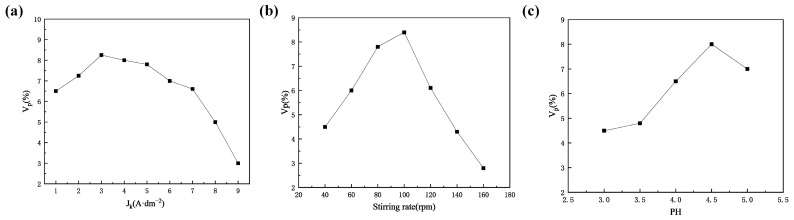
(**a**). Current density versus volume fraction of Co in the plated layer (**b**). Stirring rate versus volume fraction of Co in the plated layer (**c**) pH versus Co content in the plated layer. [120].

Wu G et al. [120] fabricated cobalt–nickel–Al_2_O_3_ composite coatings using a composite co-deposition technique by incorporating Al_2_O_3_ particles into an amino sulfonate electrolyte system. The results indicated that the Al_2_O_3_ particles added significantly enhanced the composite coatings’ hardness and wear resistance. However, the co-deposition of Al_2_O_3_ led to an increase in the internal tensile stress of the cobalt–nickel–Al_2_O_3_ coatings. Figure 11a illustrates the current density effect on the volume fraction of Co in the deposited layer, showing a trend where the Co content initially increases and then decreases as the current density rises. This behavior can be attributed to a transition from an activation-controlled metal deposition process to one dominated by diffusion-controlled particle transfer. The energy required for depositing solvated and adsorbed metal ions on particle surfaces is higher than for free solvated metal ions, which likely explains the initially low co-deposition rate. At lower current densities, free metal ions preferentially deposit due to differences in activation energy. As the current density increases, this energy gap becomes less significant, allowing for greater co-deposition of Al_2_O_3_. At even higher current densities, the weak adsorption of particles onto the cathode surface can become the limiting factor, as this process is slower than metal deposition, leading to a decrease in co-deposition. The relationship between the stirring rate and Co content in the deposited layer, shown in Figure 11b, indicates that when the particle concentration is 80 g/L, the current density is 3 A/dm^2^, and the pH is 4.5; a stirring speed of 100 rpm results in the highest Al_2_O_3_ content in the deposited layer. The pH of the electrolyte was also a crucial factor influencing the outcomes. As shown in Figure 11c, the relationship between pH and Co content in the deposited layer revealed that the highest Co content was obtained at a pH of 4.5.

Elkhoshkhany N et al. [121] developed nickel-based cermet–tungsten carbide (WC) composite coatings on stainless-steel substrates using DC electroplating. To assess the coatings’ characteristics, including surface morphology, microstructure, and elemental composition, they employed various analytical techniques such as SEM, XRD, and EDX. The results indicated that the microhardness of the composite coatings increased with higher concentrations of WC and smaller grain sizes. In a 0.5 mol/L H_2_SO_4_ electrolyte, all the composite coatings demonstrated passivation behavior, with the Ni-8g/L WC coating providing the most effective corrosion protection for the stainless-steel substrate.

Ma L et al. [122] prepared nickel–cobalt–Fe_2_O_3_ composite coatings. The findings demonstrated that the co-deposition of Fe_2_O_3_ with the nickel–cobalt alloy was more pronounced under conditions of elevated Fe_2_O_3_ concentration and moderate agitation speeds. Additionally, the deposition of cobalt was enhanced with higher concentrations of CTAB (cetyltrimethylammonium bromide). As the concentration of Fe_2_O_3_ increased, more particles were embedded within the metal matrix, which contributed to the refinement of the grain structure, resulting in smaller grain sizes for the composite coatings.

Torabinejad V et al. [123] developed advanced Ni-Fe-Co multilayer coatings via electrodeposition onto plain-carbon steel substrates. The coatings were thoroughly analyzed for their microstructure, hardness, friction behavior, and wear resistance. The results indicated that by employing a specific duty cycle during the deposition process, the multilayer coatings were created with 128, 512, and 1600 individual layers, with adjacent layers alternating between two distinct chemical compositions. As the number of layers increased, there was a noticeable enhancement in both hardness and wear resistance, while the coefficient of friction decreased. Under lower loading conditions, the primary wear mechanism for the 512- and 1600-layer coatings was identified as abrasive wear.

Kumaraguru S et al. [124] investigated the corrosion resistance of nickel–cobalt–Mn oxide (NCM) composite coatings fabricated via DC electroplating. The study found that the Ni/NCM composites exhibited outstanding corrosion resistance, which was attributed to their finer grain structure, enhanced polarization resistance, and increased charge transfer resistance. As shown in Table 5, the nickel/NCM composite (5 g/L) corrosion rate was measured at 2.608 × 10^−3^ mmpy, which was roughly 20 times lower than that of mild steel (MS) and about 9 times lower than pure nickel. Additionally, the nickel/NCM composite’s corrosion performance at an NCM concentration of 5 g/L was found to be superior to that of composite coatings prepared with higher NCM concentrations of 10 g/L and 15 g/L.

Apelt S et al. [125] employed composite electrodeposition to produce coatings that incorporated Mn_3_O_4_ particles into a cobalt matrix. Their study revealed that a significant reduction in particle inclusion and current efficiency was observed when the pH dropped below 2. The concentration of Mn_3_O_4_ in the plating solution was identified as the second most important factor affecting particle deposition, with the agitation rate following closely in terms of influence.

**Figure 12:**
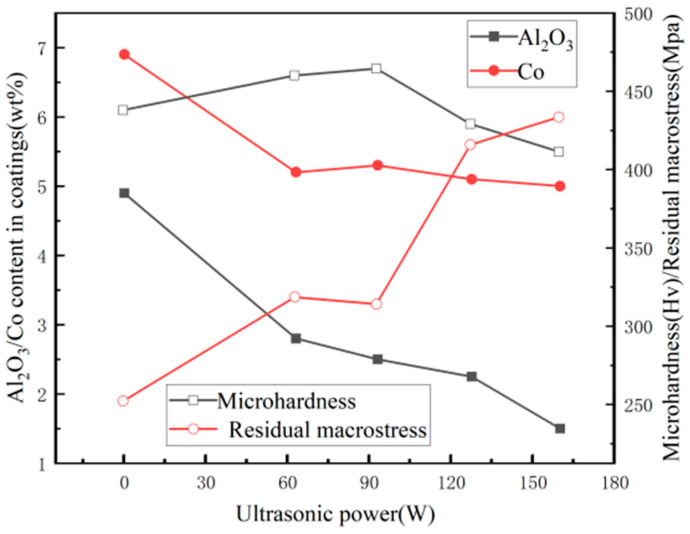
Effect of ultrasonic power on Al_2_O_3_/Co content, microhardness, and macroscopic residual stresses in composite plating [126].

Chang L M et al. [126] employed a pulsed reverse current (PRC) technique combined with ultrasonic assistance to produce nickel–cobalt/Al_2_O_3_ composite coatings. The results, presented in Figure 12, demonstrated that increasing the ultrasonic power led to a decrease in the Al_2_O_3_ particle content within the coating, along with a reduction in cobalt concentration. Despite these changes, the cobalt content showed a tendency to remain relatively stable, indicating a complex interaction between ultrasonic power and the deposition process that influences the material’s overall composition. The residual macro stress also increased with higher ultrasonic power. Initially, the microhardness of the composite layer increased, but then it declined sharply. This was attributed to the fact that the microhardness of the composite coating not only depended on the Al_2_O_3_ particle content but also on the dispersion of these particles within the coating. At lower ultrasonic power, the uniformity of the Al_2_O_3_ particle dispersion was the primary factor affecting the microhardness, with finer particle dispersion leading to higher microhardness. At higher levels of ultrasonic power, the reduced incorporation of Al_2_O_3_ particles, combined with increased particle collisions, led to the agglomeration of the particles. The clustering of Al_2_O_3_ particles not only affected the coating’s microstructure but also diminished its ability to resist deformation, ultimately weakening its mechanical properties.

Shi L et al. [127] fabricated nickel–cobalt/MoS_2_ nanocomposites coatings using the composite electrodeposition technique. The results revealed that incorporating MoS_2_ nanoparticles into the electrolyte caused a shift in the reduction potential of the nickel–cobalt alloy coatings toward more negative values. However, the co-deposition of MoS_2_ did not significantly alter the nickel–cobalt alloy coatings’ electrodeposition behavior. Despite this, the presence of MoS_2_ in the coating substantially influenced its surface morphology and microstructure. The MoS_2_ particles were uniformly distributed throughout the nickel–cobalt matrix, which played a key role in enhancing the composite coatings’ tribological performance, improving wear resistance and reducing friction.

**Figure 13:**
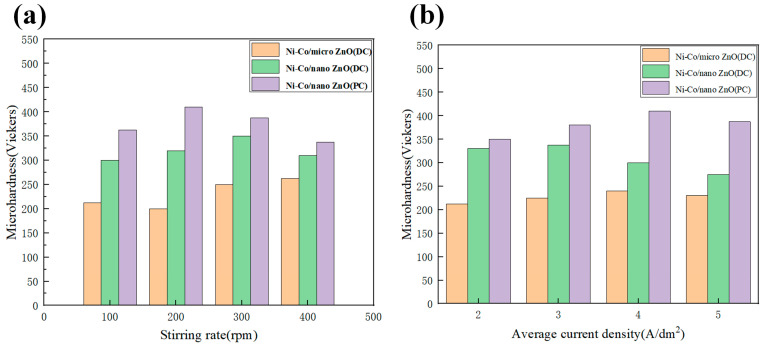
(**a**) Effect of stirring rate on microhardness of nickel–cobalt/ZnO plated layer. (**b**) Effect of average current density on microhardness of nickel–cobalt/ZnO plated layer [128].

Imanian Ghazanlou S et al. [128] developed nickel–cobalt nanocomposite coatings reinforced with ZnO particles and investigated their structural properties by employing both PC and DC electrodeposition techniques. The research demonstrated that transitioning from DC to PC electrodeposition, coupled with a reduction in the size of the ZnO particles from the micrometer to the nanometer scale, led to significant improvements in the surface characteristics of the coatings. Specifically, the surface became smoother and the friction coefficient was reduced, contributing to enhanced wear resistance. Additionally, the refined microstructure and the distribution of ZnO nanoparticles within the nickel–cobalt matrix resulted in better corrosion protection. Additionally, the change in ZnO particle size and the electrodeposition method altered the wear mechanism and the wear surface topography. The relationship between the stirring rate and microhardness of the plated layer for different cases is shown in Figure 13a, and Figure 13b displays the relationship between average current density and microhardness; both demonstrate that nano-sized particles are more effective in enhancing microhardness than micrometer-sized particles under similar conditions, materials, and classified according to the introduced reinforcing phases.

Choudhary R K et al. [129] employed a pulsed DC electrodeposition technique to fabricate Zn–nickel–cobalt composite coatings. The study revealed that the coatings were predominantly composed of zinc, with only small amounts of cobalt and nickel incorporated into the structure. It was observed that reducing the pulse frequency of the current led to an increase in the concentrations of cobalt and nickel in the coatings. While the pulse frequency had little effect on the surface morphology of the Zn–nickel–cobalt coatings, a lower pulse frequency was found to significantly improve the corrosion resistance of the plated layer.

### 4.2. Non-Metallic Substances

Arzumanova A V et al. [130] employed nanocomposite electrodeposition to develop a chloride-based electrolyte tailored for producing nickel–cobalt–silicon oxide coatings with superior wear resistance. The results showed the silicon oxide particles added to the coatings significantly enhanced their protective capabilities, offering improved wear resistance and greater durability. Wang Liping et al. [131] employed composite electrodeposition to fabricate nano-diamond-enhanced nickel–cobalt alloy coatings and investigated their wear and friction resistance. The findings showed an increase in the hardness of the nanocomposite coatings compared to pure nickel–cobalt alloy coatings. This enhancement was largely due to the nano-diamond particles’ uniform distribution within the coating matrix, which contributed to strengthening the material by impeding plastic deformation. The inclusion of high-hardness nano-diamond particles in the nickel–cobalt alloy matrix significantly improved the coating’s ability to resist sliding wear. Under optimal deposition conditions, the nanocomposite coating wear volume was reduced to about one-third of that observed for the standard nickel–cobalt alloy coating. In a related study, Xu W. et al. [132] developed a nickel–cobalt/diamond composite coating using a buried sand composite electrodeposition technique and explored the diamond particle size’s impact on the coating’s microstructure, densification, microhardness, and tribological properties. Their results revealed that as the diamond particle size increased, both the composite coating density and hardness decreased. The coatings exhibited maximum density (0.9) and hardness (950 HV) when the diamond particles were in the range of 6 to 12 μm. Furthermore, the wear loss was lower as the diamond particle size increased, with the least wear observed for particles between 125 and 150 μm. These studies underscore the positive impact of the incorporation of diamond particles into coatings, emphasizing the importance of particle size and distribution in optimizing both mechanical and wear-resistant properties.

Shi L et al. [133] developed nickel–cobalt–carbon nanotube (CNT) composite coatings through electrodeposition using a nickel–cobalt plating bath that incorporated carbon nanotubes. Their study revealed that the CNTs introduction into the electrolyte led to a shift in the nickel–cobalt alloy reduction potential toward more negative values. Despite this change, the presence of CNTs did not significantly affect the nickel–cobalt alloy’s overall electrodeposition process. The composite coatings demonstrated notable improvements in both microhardness and elastic modulus compared to pure nickel–cobalt coatings. This uniform distribution of CNTs contributed to the strengthening of the alloy matrix, significantly boosting its wear resistance and overall durability.

**Figure 14:**
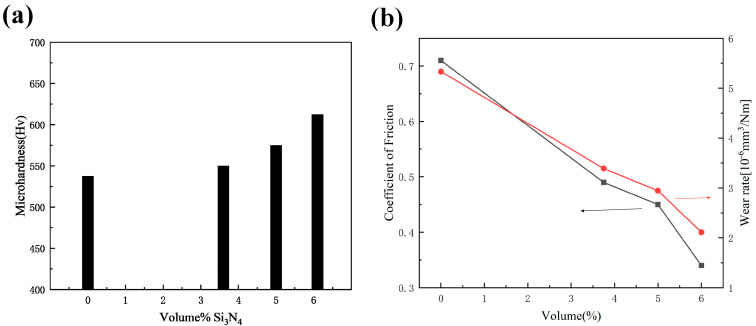
(**a**) Variation in microhardness of nickel–cobalt/Si_3_N_4_ composite coatings with nano-Si_3_N_4_ content. (**b**) Variation in friction coefficient and wear rate with volume fraction of nano-Si_3_N_4_ in composite coatings [134].

Shi L et al. [134] explored the fabrication of nickel–cobalt/Si_3_N_4_ nanocomposite coatings via electrodeposition, using a plating bath containing Si_3_N_4_ nanoparticles with an average size of about 20 nm. The study showed that increasing the concentration of Si_3_N_4_ in the electrolyte led to a shift in the cathodic polarization potential of the composite solution to more negative values. The introduction of Si_3_N_4_ particles also caused significant changes in the coatings’ surface characteristics. While the pure nickel–cobalt coatings exhibited acicular microcrystalline structures, the composite coatings that included Si_3_N_4_ demonstrated a more granular texture, with finer and denser particles. As the Si_3_N_4_ concentration increased, the morphology evolved from fibrous to more granular, with higher concentrations leading to slight aggregation of the particles. Despite this, the Si_3_N_4_ particles remained evenly distributed within the nickel–cobalt matrix, which significantly improved both the microhardness and tribological performance of the coatings. As seen in Figure 14a,b, increasing the amount of Si_3_N_4_ nanoparticles resulted in a rise in the coating’s microhardness, while simultaneously lowering both the coating’s friction coefficient and the coating’s wear rate. This improvement is attributed to the grain refinement and strengthening effects caused by the uniform dispersion of Si_3_N_4_ particles within the coating, enhancing its mechanical and wear resistance properties.

Imanian Ghazanlou S et al. [135] fabricated nickel–cobalt/SiO_2_ nanocomposite coatings. The coatings were analyzed using XRD and SEM to evaluate their microstructure and surface characteristics. It was found the nickel–cobalt/SiO_2_ nanocomposite coatings’ microhardness increased when the plating bath temperature and pH values were raised. On the other hand, when DC deposition was used, there was a noticeable decrease in hardness. Additionally, substituting SiO_2_ nanoparticles with larger SiO_2_ microparticles resulted in a reduction in microhardness. Tests on friction and wear resistance showed that the use of pulsed electrodeposition, combined with SiO_2_ nanoparticle inclusion, significantly reduced the friction coefficient and improved the coatings’ wear resistance. Furthermore, corrosion resistance measurements, including anodic polarization in a 3.5 wt.% NaCl solution, suggested the combination of SiO_2_ nanoparticles and pulsed current deposition enhanced the coating’s resistance to corrosion. These results highlight the important role that deposition parameters, such as plating bath conditions and current type, play in enhancing the composite coating’s performance.

Ghazanlou S I et al. [136] employed the pulsed electrodeposition technique to fabricate nickel–cobalt/SiO_2_ nanocomposite coatings on steel substrates. The optimal deposition parameters were found to be a 200 rpm stirring speed, a pH value of 4.6, and a 20 g/L SiO_2_ concentration. Under these conditions, the addition of SiO_2_ particles to the nickel–cobalt matrix resulted in coatings that exhibited significantly improved microhardness, enhanced corrosion resistance, and lower wear mass loss compared to pure nickel–cobalt coatings. Furthermore, the composite coatings demonstrated a reduced coefficient of friction. These improvements suggest that the incorporation of SiO_2_ nanoparticles into the plating process effectively enhances both the resulting coatings’ mechanical and tribological performance.

IMANIANGHAZANLOUS et al. [137] prepared nickel–cobalt/SiO_2_ composite coatings on steel substrates by employing both DC and pulsed electrodeposition techniques. To analyze the properties of the coatings, various characterization techniques were used, including XRD, FESEM, EDX, and X-ray elemental mapping, allowing for a detailed examination of the coatings’ phase composition, surface morphology, and elemental distribution. The findings revealed that the composite coatings’ surface characteristics were significantly affected by the electrodeposition conditions. At higher current densities, the coatings exhibited a rough, irregular texture with spherical structures, while at lower densities, the surface became smoother and more ordered, featuring finer spheres. Moreover, when the duty cycle was increased to 50%, the microhardness of the coatings improved, and the grain size was reduced as the average current density increased from 2 A/dm^2^ to 6 A/dm^2^. However, when the duty cycle surpassed 50%, resulting in a current density of 8 A/dm^2^, a reduction in microhardness and an increase in grain size was observed.

Additionally, coatings produced using pulsed current deposition showed higher microhardness compared to those formed using a DC method. The study determined that the optimal pulse frequency for achieving the best coating properties was approximately 25 Hz. These results underscore the importance of electrodeposition parameters such as current density and duty cycle, which significantly influence the microstructure and overall performance of nickel–cobalt/SiO_2_ composite coatings.

Akbarpour M R et al. [138] fabricated nickel–cobalt/graphene nanocomposite coatings using pulsed reverse current (PRC) electrodeposition in a solution containing both nickel–cobalt and graphene (Gr) nanoparticles. The research focused on understanding how varying the concentration of graphene affected the resulting coatings’ hardness and morphological, microstructural, and electrochemical properties. The results demonstrated that the inclusion of graphene in the electrolyte had a notable effect on both the composite coating composition and the composite coating microstructure. As the graphene concentration increased in the bath, there was a corresponding rise in the cobalt content within the nickel–cobalt alloy coatings. The coatings produced under PRC conditions exhibited a smooth, tightly packed surface with an average grain size between 8 and 10 nm, revealing a solid solution of Ni (Co). This uniform distribution of graphene particles within the nickel–cobalt matrix contributed to enhanced mechanical and electrochemical characteristics.

An electrochemical analysis showed that increasing the graphene content improved the coating’s corrosion resistance, although this trend plateaued when the graphene concentration exceeded 0.8 g/L. Beyond this concentration, the electrochemical properties declined, likely due to particle aggregation, which negatively impacted the uniformity and effectiveness of the coating. Additionally, the composite coatings’ microhardness increased significantly with higher graphene concentrations, reaching a remarkable 610 HV, compared to just 458 HV in the pure nickel–cobalt coatings. This study underscores the potential of incorporating graphene into nickel–cobalt coatings to boost both their mechanical and electrochemical properties, offering a promising approach for creating advanced coatings with superior performance in various industrial applications.

### 4.3. Silicon Carbide (SiC)

To explore the relationship between the structure and properties of composite materials, it is evident that introducing second-phase ceramic particles into nickel–cobalt alloy coatings via electrodeposition can provide unique characteristics that differentiate them from standard electroplated alloys. This method exploits the benefits of ceramic particles, which enhance several critical properties such as hardness, wear resistance, corrosion protection, and heat tolerance.

For instance, incorporating Al_2_O_3_ particles into the composite coatings enhances their hardness and wear resistance. The addition of these particles refines the coating microstructure, improving its durability and reducing its susceptibility to abrasion, making it ideal for applications that involve high levels of mechanical stress or friction [117,121,127]. Similarly, the inclusion of Si_3_N_4_ particles has been shown to improve not only the microhardness but also the coating’s frictional properties and the coating’s wear rate. This results in longer-lasting coatings with lower friction coefficients, making them more efficient in reducing wear over time [134].

Adding ZrO_2_ particles [119] to the coating composition has been found to significantly boost both the coating’s corrosion resistance and microhardness. ZrO_2_ is known for its ability to form a protective and stable layer on the coating, which improves its ability to resist aggressive chemical environments. This feature is especially beneficial for coatings used in industries where exposure to harsh chemicals is common, such as in chemical processing or marine applications.

In addition, SiC in ceramic particles has attracted widespread attention due to its excellent chemical stability, high microhardness, good wear resistance, corrosion resistance at high temperatures [139,140,141], outstanding creep resistance [142], oxidation resistance [143], and radiation resistance [144,145].

Bakhit B et al. [146] prepared nickel–cobalt alloy and nickel–cobalt/SiC nanocomposite coatings. Their findings revealed significant information on the behavior of SiC nanoparticles within the composite coatings under various deposition conditions. The investigation showed that the coatings, whether composed of pure nickel–cobalt or the nickel–cobalt/SiC composite, maintained an FCC crystal structure throughout the experimental range of current densities. Notably, this phase structure remained stable even as the current density was altered, indicating that the SiC nanoparticle integration into the nickel–cobalt matrix did not disrupt the crystalline orientation of the metal. However, the incorporation of SiC nanoparticles was observed to follow a distinct trend depending on the applied current density. Initially, as the current density increased, the concentration of SiC nanoparticles in the coatings also increased, peaking at a current density of 3 A/dm^2^. Beyond this point, further increases in current density caused the SiC nanoparticle concentration to decrease. This was explained by the competition between the metal ion reduction rate and the suspended SiC particle incorporation. At lower current densities, the rate of metal ion reduction dominated, leading to fewer SiC particles being incorporated into the coating. As the current density increased, the incorporation rate of the nanoparticles also increased, but once the current density exceeded a certain threshold, the metal ion reduction rate outpaced the SiC particle incorporation, resulting in a lower concentration of SiC in the coating. In addition to these observations, EDS analysis indicated that the nickel and cobalt peak intensity increased with higher current densities. This increase further reinforced the notion that higher current densities promote metal ion reduction, leading to an increased presence of Ni and Co in the coatings, while the SiC nanoparticle concentration decreased as a result of the competitive nature of the deposition process.

In conclusion, the study by Bakhit B et al. provides valuable insights into the deposition process of nickel–cobalt/SiC nanocomposite coatings, highlighting the current density’s influence on both the structural properties and the distribution of SiC nanoparticles. The ability to fine-tune the incorporation of SiC nanoparticles by adjusting current density could lead to the development of coatings tailored for specific high-performance applications where enhanced mechanical properties are required.

Dai PQ et al. [147] investigated the fabrication of nanocrystalline nickel–cobalt alloy and nickel–cobalt/SiC nanocomposite coatings through pulsed electrodeposition. Their research focused on how the addition of nano-sized silicon carbide (SiC) particles influences the coating’s microstructure and the coating’s performance. The study revealed that incorporating SiC nanoparticles resulted in a significant reduction in the nickel–cobalt/SiC composite coatings’ grain size. This grain refinement plays a critical role in improving the hardness of the coatings. The smaller grain structure, facilitated by the SiC particle’s uniform dispersion, helps to strengthen the coating by hindering dislocation motion. As the content of SiC nanoparticles increased, the coating’s microhardness also improved, surpassing that of the pure nickel–cobalt alloy coatings. This enhancement in hardness was primarily attributed to the dual effects of grain refinement and the strengthening effect of the dispersed SiC particles in the matrix. In addition to mechanical strengthening, the presence of SiC nanoparticles also contributed to an improvement in the composite coatings’ corrosion resistance. Specifically, the corrosion potential became more positive as the SiC nanoparticle concentration increased, signifying improved passivation and protective properties. Furthermore, the corrosion current was lower for the nanocomposite coatings, indicating less corrosion activity and better long-term protection for the underlying substrate. The SiC nanoparticles are embedded within the nickel–cobalt matrix, filling microvoids and pores in the coating, which helps reduce pathways for corrosive elements to penetrate. This nanoparticle inclusion contributes to a more compact formation and continuous surface layer that impedes the ingress of harmful agents. Moreover, the SiC particles promote the passivation of the matrix, improving its overall resistance to corrosion. The SiC nanoparticles also act as barriers, preventing the initiation and propagation of corrosion pits that typically occur in less protective coatings. From the electrochemical results and structural analysis, it is clear that the nickel–cobalt/SiC nanocomposite coatings offer both significantly higher hardness and improved corrosion resistance compared to pure nickel–cobalt alloys.

Shi L et al. [148] prepared nickel–cobalt nanocomposite coatings via electrodeposition with the inclusion of SiC nanoparticles in a plating solution. They observed that increasing the SiC particle concentration in the electrolyte led to a shift in the cathodic polarization potential to more negative values, indicating enhanced electrochemical properties of the composite electrolyte. As the amount of SiC in the solution was increased, the microhardness of the composite coatings improved, reflecting the strengthening effect of the fine ceramic particles embedded in the nickel–cobalt matrix. This enhancement in hardness can be attributed to the dispersion-hardening mechanism, where the SiC particles act as obstacles to dislocation motion, thus increasing the resistance to deformation. Moreover, the composite coating wear resistance was enhanced with higher SiC content. The uniformly distributed SiC nanoparticles within the matrix contributed to improved resistance to abrasive wear, which is critical in applications involving mechanical contact or friction. The composite coatings’ wear properties were further supported by the fact that SiC, known for its high hardness and durability, reinforces the overall toughness of the coating. In addition to mechanical improvements, the composite coatings’ corrosion resistance also showed an increase with rising SiC concentration. This result aligns with previous studies, such as that by Q. Dai, which demonstrated that the incorporation of ceramic particles like SiC into metallic matrices can enhance corrosion resistance. The SiC particles’ uniform distribution in the nickel–cobalt matrix helps to form a more stable and denser protective layer, which reduces the corrosion initiation likelihood and corrosion pit formation. The composite coating morphology also varied with changes in SiC concentration. At higher SiC contents, the coatings exhibited a more refined, uniform structure, with the SiC nanoparticles evenly distributed throughout the metal matrix. This distribution not only contributed to better mechanical properties but also facilitated the formation of a more cohesive protective layer on the coating surface.

Srivastava Sr M et al. [149] discovered that increasing the doping of nano-SiC particles in Ni/nickel–cobalt composites led to a rise in microhardness. In composites reinforced with micron-sized SiC particles, the cobalt content was found to increase. The volume loss in nickel–cobalt composites was less than in pure Ni composites. Furthermore, as shown in Table 6, the wear performance of nickel–cobalt composites reinforced with micron-sized SiC was superior to that of those reinforced with nano-SiC particles.

Shi Lei et al. [150] developed Ni/Co-SiC nanocomposite coatings. The incorporation of nano-SiC particles into the plating solution led to a significant refinement in the grain structure of the coatings. Initially characterized by acicular grains, the coating morphology transformed into a more uniform granular structure as the SiC concentration in the electrolyte increased. This refinement of the microstructure was primarily attributed to the SiC particle dispersion-strengthening effect, which hindered the growth of metal grains during electrodeposition.

Kamel M M et al. [151] developed nickel–cobalt/SiC composite coatings. The results revealed that the composite coatings exhibited an FCC crystal structure, characteristic of nickel–cobalt alloys. This structure was consistent regardless of the SiC content, confirming that the SiC particle incorporation did not alter the fundamental crystallographic phase of the alloy. However, the addition of SiC particles influenced other properties, particularly the deposited coating thickness. As the weight percentage of SiC increased in the bath solution, the thickness of the coatings grew progressively. This suggests that the SiC nanoparticles’ presence may affect the rate of deposition, possibly by influencing the electrolyte composition or the electrochemical kinetics of the process.

In terms of performance, the study found that the nickel–cobalt/SiC composite coatings’ corrosion resistance improved significantly with higher SiC content. The SiC particle incorporation likely enhanced the coatings’ ability to protect the underlying steel substrate by filling voids and pores in the deposited layer, which can act as sites for corrosion initiation. The added SiC particles also served as a barrier to corrosion, preventing the ions’ diffusion through the coating, thereby enhancing the overall corrosion protection. Additionally, the composite coatings’ microhardness and scratch resistance were found to improve with increasing concentrations of SiC particles. The hard ceramic nature of SiC particles contributes to the increased hardness of the composite coatings, providing better wear resistance and surface durability. The incorporation of SiC into the coating matrix led to a more robust material capable of withstanding mechanical stress and friction, which is critical for industrial applications requiring wear-resistant coatings.

Ababsa A et al. [152] synthesized nickel–cobalt nanocomposite coatings via electrodeposition in modified Watts baths. The findings indicated that the co-deposition of uniformly distributed SiC particles led to a significant enhancement in the nickel–cobalt deposits’ ultimate tensile strength. The presence of SiC nanoparticles also facilitated a shift toward a more ductile fracture mode, especially at higher strain rates, whereas the coatings without SiC exhibited more brittle fracture characteristics. This mixed fracture behavior, a combination of ductile and brittle failure, suggests that the SiC particles play a key role in modifying the mechanical properties of the nickel–cobalt matrix, making it more resilient under certain stress conditions.

In another study, Yang Y et al. [153] investigated the nucleation and growth mechanisms of nickel–cobalt–SiC nanocomposite coatings on carbon steel substrates. Their results showed that in the initial stages, the deposition followed a combination of transient and progressive mechanisms when no cathodic overpotential was applied. However, at higher cathodic overpotentials, the deposition process was dominated by a transient mechanism. Notably, SiC nanoparticles added to the electrolyte significantly reduced the overall electrodeposition efficiency compared to the deposition of pure nickel–cobalt coatings. SiC particles hindered metal matrix nucleation and metal matrix growth, likely due to their larger size and inability to fully integrate into the deposition structure, thereby slowing down the overall deposition rate.

Similarly, Bahadormanesh B et al. [154] explored nickel–cobalt/SiC coating nanocomposite electrodeposition with varying concentrations of SiC particles. Their results demonstrated that the highest concentration of SiC incorporated into the coatings was achieved under the following conditions: a current density of 4 A/dm^2^, a SiC concentration of 40 g/dm³, and a stirring speed of 480 rpm. The presence of SiC particles also facilitated the nickel and cobalt ions’ anomalous co-deposition, which contributed to enhancing the overall electrodeposition process. This anomalous co-deposition is thought to occur due to the specific interaction between the metal ions and the SiC nanoparticles, improving the uniformity and quality of the composite coatings.

These studies collectively highlight the impact of SiC nanoparticles on the properties of nickel–cobalt alloy coatings. The incorporation of SiC not only enhances the mechanical properties, such as tensile strength and hardness, but also influences the deposition process and the microstructural development of the coatings. Despite the challenges associated with SiC’s impact on the deposition rate and efficiency, its presence in the matrix significantly improves the wear resistance and overall durability of the resulting nanocomposite coatings.

The current research status of composite electrodeposition methods for nickel–cobalt composite materials is shown in Table 7. The effects of adding different types of substances on the properties of nickel–cobalt composite materials vary.

## 5. Summary and Outlook

This paper provides an overview of the current state of research into the preparation of Ni-Co system coatings via the composite electrodeposition method. The findings highlight that several factors, including the concentration of the plating solution, current density, bath temperature, pH, and the concentration of incorporated particles, have significant effects on both the surface morphology and the properties of the resulting composites. Research has shown that cobalt content has a significant impact on the properties of Ni-Co alloy coatings. As the cobalt content increases, the hardness and wear resistance of the coating first increase and then decrease. When the cobalt content is between 20 and 50% and 60 and 80%, the coating hardness reaches a higher value. However, excessive cobalt content can cause the coating to become brittle, affecting its corrosion resistance. Using pulsed current can obtain more uniform and dense coatings than using direct current. A high current density can lead to a decrease in the cobalt content in the coating, while low current density can help improve the uniformity and quality of the coating. Further, the current density and pulse parameters can be optimized to achieve more uniform particle distribution and higher coating performance. At the same time, new electrodeposition techniques can be explored, such as high-frequency pulse electrodeposition, to further improve the coating quality. The pH value has a significant impact on the deposition process and material quality of Ni-Co composite materials. During the composite electrodeposition process, maintaining a pH value of around 4.5 is considered an ideal condition. Within this pH range, the deposition efficiency of cobalt is high, and it can effectively avoid excessive particle aggregation, thereby obtaining uniformly distributed composite materials. At lower pH values, the reduction reaction rate of metal ions is faster, but the adsorption and co-deposition efficiency of particles is lower; at higher pH values, although the adsorption capacity of particles is enhanced, it may lead to an increase in the precipitation reaction of metal ions, affecting the uniformity and density of the coating. During the composite electrodeposition process, when the introduced particle size is small, such as nano-sized SiC, Al_2_O_3_, or ZrO_2_ particles, agglomeration is prone to occur. This agglomeration is mainly due to the strong van der Waals forces and electrostatic forces between particles, which make it difficult for the particles to be uniformly dispersed in the solution. Particle agglomeration seriously affects the uniformity and performance of the coating. Agglomerated particles may cause pores or defects in the material, reducing the hardness, wear resistance, and corrosion resistance of the coating. To reduce particle agglomeration, methods such as optimizing particle size, adding dispersants, and controlling electrodeposition parameters can be adopted. In terms of current density, research has shown that a current density within the range of 2–6 A/dm^2^ is more suitable, as high or low current densities can affect the quality and performance of materials. In terms of temperature, the temperature of the plating solution has a significant impact on the deposition rate and crystallinity of the material. The suitable temperature range is 30–50 °C, within which higher deposition rates and better material quality can be achieved.

Despite the promising potential of nickel–cobalt composite electrodeposition, this technique remains in the developmental phase and has not yet reached full maturity. Several challenges still need to be addressed before it can be widely applied in industrial settings. For instance, issues related to the fracture toughness of the coatings remain unsolved. Residual stresses within the electrodeposited coatings can cause premature failure under operating conditions, and methods for testing and optimizing these stresses need further research. Additionally, when the reinforcing particles are introduced into the bath at too small a size, they may aggregate, leading to poor dispersion and a non-uniform distribution within the coating. Such aggregation can undermine the composite’s performance.

For nickel–cobalt nanocomposites, future research can focus on optimizing the electrodeposition process parameters to control the crystal structure, particle size, distribution, and morphology of the materials, thereby improving their mechanical, chemical, and electrochemical properties. In addition, by improving the composition of the electrolyte (such as adding different additives or surfactants), the quality and performance of the deposited materials can be further enhanced. The selection of additives can regulate the microstructure of nanocomposites, optimize their surface morphology, and thereby improve their conductivity, corrosion resistance, and catalytic activity. In terms of nanostructures and surface modification, by controlling the electrodeposition process we can further explore the nanostructure design of nickel–cobalt nanocomposites while studying surface modification techniques (such as coating, doping, or functionalization) to improve the corrosion resistance, oxidation resistance, and other properties of nickel–cobalt nanocomposites. In terms of interface effects and material characterization, future research should focus on the interface interactions between nickel–cobalt-based materials and other components (such as dopants, auxiliary materials, etc.), and understanding the mechanisms of the interface in electrochemical reactions, catalytic reactions, etc. Nickel–cobalt nanocomposites have shown broad prospects in various non-silicon fields such as energy storage and conversion, sensors, etc., due to their unique electrical, chemical, and physical properties.

At present, the preparation of nickel–cobalt composites via electrodeposition is largely confined to laboratory-scale experiments, and scalability to industrial mass production remains a significant hurdle. To address these challenges, further exploration is needed in areas such as residual stress management and the prevention of particle agglomeration. Additionally, research into scaling the process for industrial applications, particularly in fields such as non-silicon MEMSs (micro-electro-mechanical systems), holds great potential. Overcoming these obstacles will pave the way for more widespread use of composite electrodeposition in industrial applications, offering enhanced material performance for a range of surface modification needs.

## Figures and Tables

**Table 1:** Comparison of the methods for preparing nickel–cobalt alloys.

Comparison Aspect	Sol–Gel Method	Phase Reduction	Electroplating	Mechanical Alloying	Composite Electrodeposition
Preparation method	Sol–gel process using metal alkoxides	Reduction using a metal salt and reducing agent	Electrochemical deposition onto a substrate	Ball milling and mechanical energy input from	Electrochemical co-deposition with additives
Equipment	Furnace for heat treatment	Reaction vessel, stirring	Power supply, substrate	ball mill, protective atmosphere	Electroplating setup
Cost	Moderate	Moderate to high	Low	High	Moderate
Environmental impact	Low	Moderate	Moderate	Low	Moderate

**Table 2:** Correspondence between Co^2+^ content in the plating solution and Co elemental content in the plated layer [87].

Sample	Ni 20%Co	Ni 40%Co	Ni 60%Co	Ni 80%Co
Co^2+^ concentration percentage (%)	2.5	9.2	12.5	28.7
Co atomic percentage (%)	20.14	40.33	59.97	80.14

**Table 3:** Grain sizes of various Ni-Co alloy, pure nickel, and pure cobalt coatings [93].

Coating	Crystallite Size (nm)	Coating	Crystallite Size (nm)
Pure Ni	23.2	Nickel–70% cobalt	17.6
Nickel–20% cobalt	23.1	Nickel–80% cobalt	17.6
Nickel–50% cobalt	11.5	Pure Co	17.6

**Table 4:** The influence of different factors on the experimental results.

Process Parameter	Effect on Coating Properties	Specific Impact
Cobalt Content	Alloy composition	Higher cobalt content increases hardness and wear resistance. Excessive cobalt may make the coating brittle and affect corrosion resistance. Affects electrical conductivity, oxidation resistance, and magnetism.
Current Density	Coating quality, uniformity, and thickness	High current density leads to thicker coatings but may cause unevenness. Low current density results in more uniform coatings. Very low current density may result in thin coatings with poor durability.
Current Type	Deposition rate and coating performance	DC current yields thicker, uniform coatings. Pulse current improves crystal structure, hardness, and corrosion resistance. Different current types affect coating morphology and microstructure.
pH Value	Crystallization behavior and composition	Low pH (acidic) increases brittleness and solubility of the coating. High pH (alkaline) may hinder cobalt deposition, leading to poor coating quality. Moderate pH helps achieve uniform coatings with controlled composition.
Temperature	Deposition rate and composition stability	High temperature accelerates deposition but may lead to coarse grains, affecting mechanical properties. Low temperature reduces deposition rate and can cause uneven coatings. Optimal temperature improves microstructure stability, hardness, and corrosion resistance.

**Table 5:** Corrosion results of Ni/NCM composite coatings in 3.5% NaCl solution [124].

NCM	J_corr_ (A·cm^−2^)	E_corr_ (mV vs. SCE)	R_p_	Corrosion Rate (CR)		Rs	Rct	Thickness
			(kΩcm^2^)	(mpy)	(mmpy)	(Ωcm^2^)	(kΩcm^2^)	(μm)
MS	5.350 × 10^−6^	−525.2	1.357	2.4446	6.210 × 10^−2^	10.46	0.916	-
Ni	2.163 × 10^−6^	−475.7	3.042	0.9180	2.332 × 10^−2^	11.80	2.106	50.04
5	2.419 × 10^−7^	−302.9	38.670	0.1026	2.608 × 10^−3^	10.61	90.951	58.13
10	2.325 × 10^−6^	−843.7	4.118	0.9868	2.507 × 10^−2^	8.24	31.453	60.89
15	6.603 × 10^−7^	−759.3	12.387	0.2802	7.118 × 10^−3^	9.11	23.845	62.82

**Table 6:** Grain size of various Ni-Co alloy, pure nickel, and pure cobalt coatings [149].

Composite	Wear Coefficient	Coefficient of Friction (avg.)
Ni-SiC nano	8.33 × 10^−6^	0.835
Ni-SiC micron	2.42 × 10^−5^	0.707
Ni-rich Ni-Co composite		
Ni-Co-SiC nano	3.35 × 10^–6^	0.834
Ni-Co-SiC micron	1.31 × 10^−6^	0.801
Co-rich Ni-Co composite		
Ni-Co-SiC nano	1.77 × 10^−6^	0.821
Ni-Co-SiC micron	1.36 × 10^−6^	0.847

**Table 7:** The current research status of composite electrodeposition methods for nickel–cobalt composite materials.

Research Variable	Metals or Metal Compounds	Non-Metal Substances	Silicon Carbide
Metal/Compound Types	Common metals include nickel, cobalt, copper, silver, and aluminum, and compounds may involve oxides or nitrides.	Non-metallic substances like nitrides, borides, carbons, and fluorides are often used in composite materials	Silicon carbide, a common ceramic material, is used to enhance physical properties when combined with nickel–cobalt metals.
Advantages	Good electroplating properties, forming uniform coatings. Enhanced electrochemical performance, such as conductivity and corrosion resistance.	Improved high-temperature resistance and wear resistance. Enhanced mechanical strength and corrosion resistance.	Increased hardness, wear resistance, and thermal expansion resistance of the nickel–cobalt composite material. Enhanced oxidation resistance and chemical stability of the material.

## Data Availability

Data available on request from the authors.

## References

[1] Luo J.K., Flewitt A.J., Spearing S.M., Fleck N.A., Milne W.I. (2004). Young’s modulus of electroplated Ni thin film for MEMS applications. Mater. Lett..

[2] Allameh S.M., Lou J., Kavishe F., Buchheit T., Soboyejo W.O. (2004). An investigation of fatigue in LIGA Ni MEMS thin films. Mater. Sci. Eng. A.

[3] Yang Y., Imasogie B.I., Allameh S.M., Boyce B., Lian K., Lou J., Soboyejo W.O. (2007). Mechanisms of fatigue in LIGA Ni MEMS thin films. Mater. Sci. Eng. A.

[4] Teh K.S., Cheng Y.T., Lin L. (2005). MEMS fabrication based on nickel-nanocomposite: Film deposition and characterization. J. Micromech. Microengin..

[5] Cohen A.L., Frodis U., Tseng F.G., Zhang G., Mansfeld F., Will P.M. (1999). EFAB: Low-cost automated electrochemical batch fabrication of arbitrary 3D microstructures. Micromach. Microfabr. Process Technol. V SPIE.

[6] Khan F., Zhu Y., Lu J., Pal J. (2015). MEMS-based tunable meander inductor. Electron. Lett..

[7] Huang M., Ruoff R.S. (2020). Growth of single-layer and multilayer graphene on Cu/Ni alloy substrates. Acc. Chem. Res..

[8] Deng J., Chen C., Liu X., Li Y., Zhou K., Guo S. (2021). A high-strength heat-resistant Al–5.7Ni eutectic alloy with spherical Al_3_Ni nano-particles by selective laser melting. Scr. Mater..

[9] Kong F.P., Ren Z.H., Banis M.N., Du L., Zhou X., Chen G., Zhang L., Li J., Wang S., Li M. (2020). Active and stable Pt–Ni alloy octahedra catalyst for oxygen reduction via near-surface atomical engineering. ACS Catal..

[10] Bhat R.S., Shetty S.M., Kumar N.V.A. (2021). Electroplating of Zn-Ni alloy coating on mild steel and its electrochemical studies. J. Mater. Eng. Perform..

[11] Kumaraswamy J., Kumar V., Purushotham G., Suresh R. (2021). Thermal analysis of nickel alloy/Al_2_O_3_/TiO_2_ hybrid metal matrix composite in automotive engine exhaust valve using FEA method. J. Therm. Eng..

[12] Li Y., Zhou Y., Pan J., Liu E., Hao J., Han J. (2023). Facile Fabrication of High-White and Robust Superhydrophobic Ni/Al_2_O_3_ Composite Coating on Al Alloy. Adv. Eng. Mater..

[13] Dwivedi S.P., Sharma S. (2024). The behaviour of AZ91 magnesium-based alloy with Ni–Al_2_O_3_ reinforcement. Mater. Sci. Technol..

[14] Fahrenholtz W.G., Ellerby D.T., Loehman R.E. (2000). Al_2_O_3_–Ni composites with high strength and fracture toughness. J. Am. Ceram. Soc..

[15] Zhou Y., Sun Z.P., Yu Y., Li L., Song J.L., Xie F.Q., Wu X.Q. (2021). Tribological behavior of Ni–SiC composite coatings produced by circulating-solution electrodeposition technique. Tribol. Int..

[16] Khazrayie M.A., Aghdam A.R.S. (2010). Si_3_N_4_/Ni nanocomposite formed by electroplating: Effect of average size of nanoparticulates. Trans. Nonferrous Met. Soc. China.

[17] Lai L.Y., Wu Y.P., Yang Y., Wang H., Yang Z.Q., Ding G.F. (2019). Microfabrication and characterization of high tensile strength SiC whisker-reinforced nickel composite coatings by electrodeposition. J. Electrochem. Soc..

[18] Matsui N., Mashimo K., Egami A., Konishi A., Okada O., Tsukada T. (2002). Etching characteristics of magnetic materials (Co, Fe, Ni) using CO/NH_3_ gas plasma for hardening mask etching. Vacuum.

[19] Gu Q. (2012). Application of Cobalt in High Performance Magnetic Materials. World Nonferrous Met..

[20] Majeed A., Khan M.A., Ahmad R., Lodhi M.Y., Ahmad I. (2021). Tuning the properties of novel magnetic oxide via Co–Bi Co-substitution including theoretical background of characterization techniques. J. Supercond. Nov. Magn..

[21] Dong Y., Zhang T.L., Wang H., Liu X., Jiang C.B. (2021). Chemical synthesis and characterization of SmCo_5_/Co magnetic nanocomposite particles. Rare Met..

[22] Zhang Y.Y., Ma R., Feng S.H., Cheng L., Davies P.A., Yu P. (2020). Microstructures and magnetic properties of Fe-35% Co alloy fabricated by metal injection molding. J. Magn. Magn. Mater..

[23] Wu X., Liu X., Liao J. (2015). The Effect of WC Carbon Content on the Properties and Hard Phase Grain Size of High Cobalt Hard Alloys. Cem. Carbides.

[24] Tikkanen M.H., Taskinen A., Taskinen P. (1975). Characteristic properties of cobalt powder suitable for hard metal production. Powder Met..

[25] Klünsner T., Mitterhuber-Gressl L., Maier K., Beckstein F., Czettl C. (2024). Thermal conductivity loss in WC-Co hard metal due to high-temperature cyclic loading damage as a function of microstructure and temperature. Int. J. Refract. Met. Hard Mater..

[26] da Silva E.N., dos Santos A.A.A., do Nascimento R.M., Alves S.M., Guimarães R.S., Filgueira M. (2021). Investigation of characteristics and properties of spark plasma sintered ultrafine WC-6.4 Fe3.6Ni alloy as potential alternative WC-Co hard metals. Int. J. Refract. Met. Hard Mater..

[27] Yang Y.K., Zhang C.Q., Wang D., Nie L.P., Wellmann D., Tian Y.T., Ju J., Shen Z., Kang M.D., Zhang J.Q. (2020). Additive manufacturing of WC-Co hardmetals: A review. Int. J. Adv. Manuf. Technol..

[28] Ju J., Shen Z., Kang M., Zhang J., Wang J. (2022). On the preferential grain boundary oxidation of a Ni-Co-based superalloy. Corros. Sci..

[29] Knop M., Mulvey P., Ismail F., Radecka A., Rahman K.M., Lindley T.C., Shollock B.A., Hardy M.C., Moody M.P., Martin T.L. (2014). A new polycrystalline Co-Ni superalloy. JOM.

[30] Wei W., Xiao J.C., Wang C.F., Cheng Q., Guo F.J., He Q., Wang M.S., Jiang S.Z., Huang C.X. (2022). Hierarchical microstructure and enhanced mechanical properties of SLM-fabricated GH5188 Co-superalloy. Mater. Sci. Eng. A.

[31] Murray S.P., Pusch K.M., Polonsky A.T., Torbet C.J., Seward G.G.E., Zhou N., Forsik S.A.J., Nandwana P., Kirka M.M., Dehoff R.R. (2020). A defect-resistant Co–Ni superalloy for 3D printing. Nat. Commun..

[32] Song J.Z., Chen Y.H., Hao X.C., Wang M., Ma Y.C., Xie J.L. (2024). Microstructure and mechanical properties of novel Ni–Cr–Co-based superalloy GTAW joints. J. Mater. Res. Technol..

[33] Gao Q.Z., Jiang Y.J., Liu Z.Y., Zhang H.L., Jiang C.C., Zhang X., Li H.J. (2020). Effects of alloying elements on microstructure and mechanical properties of Co–Ni–Al–Ti superalloy. Mater. Sci. Eng. A.

[34] Liu P., Zhang R., Yuan Y., Cui C.Y., Zhou Y.Z., Sun X.F. (2020). Hot deformation behavior and workability of a Ni–Co based superalloy. J. Alloys Compd..

[35] Li J.H., Li X.H., Hu Q.Y., Wang Z.X., Zheng J.C., Wu L., Zhang L.X. (2009). Study of extraction and purification of Ni, Co and Mn from spent battery material. Hydrometallurgy.

[36] Iqbal M.Z., Faisal M.M., Ali S.R., Farid S., Afzal A.M. (2020). Co-MOF/polyaniline-based electrode material for high performance supercapattery devices. Electrochim. Acta.

[37] Zhang D., Guo X.M., Tong X.Z., Chen Y.F., Duan M.T., Shi J., Jiang C.W., Hu L.L., Kong Q.H., Zhang J.H. (2020). High-performance battery-type supercapacitor based on porous biocarbon and biocarbon supported Ni–Co layered double hydroxide. J. Alloys Compd..

[38] Zhou J.N., Yang Q.Y., Xie Q.Y., Ou H., Lin X.M., Zeb A., Hu L., Wu Y.B., Ma G.Z. (2022). Recent progress in Co–based metal–organic framework derivatives for advanced batteries. J. Mater. Sci. Technol..

[39] Sun J.L., Tian X.D., Xu C.J., Chen H.Y. (2021). Porous CuCo_2_O_4_ microtubes as a promising battery-type electrode material for high-performance hybrid supercapacitors. J. Mater..

[40] Wang H., Liu Y., Zhang J. (2023). Preparation of nitrogen doped mesoporous carbon supported cobalt catalyst and its performance in removing hydrogen rich gas CO. Power Gener. Technol..

[41] Tohidi M.M., Paymard B., Vasquez-García S.R., Fernández-Quiroz D. (2023). Recent progress in applications of cobalt catalysts in organic reactions. Tetrahedron.

[42] Dong Z.L., Jiang T., Xu B., Zhang B.S., Liu G.Q., Li Q., Yang Y.B. (2021). A systematic and comparative study of copper, nickel and cobalt-ammonia catalyzed thiosulfate processes for eco-friendly and efficient gold extraction from an oxide gold concentrate. Sep. Purif. Technol..

[43] Yong H., Wei X., Hu J., Yuan Z., Guo S., Zhao D., Zhang Y. (2021). Hydrogen storage behavior of Mg-based alloy catalyzed by carbon-cobalt composites. J. Magnes. Alloys.

[44] Paksoy A., Kurtoğlu S.F., Dizaji A.K., Altıntaş Z., Khoshsima S., Uzun A., Balcı Ö. (2021). Nanocrystalline cobalt–nickel–boron (metal boride) catalysts for efficient hydrogen production from the hydrolysis of sodium borohydride. Int. J. Hydrogen Energy.

[45] Kim J., Kim H., Han G.H., Hong S., Park J., Bang J., Kim S.Y., Ahn S.H. (2022). Electrodeposition: An efficient method to fabricate self-supported electrodes for electrochemical energy conversion systems. Exploration.

[46] Choi K.H., Kim H.S., Lee T.H. (1998). Electrode fabrication for proton exchange membrane fuel cells by pulse electrodeposition. J. Power Sources.

[47] Li X.M., Du X., Ma X.L., Wang Z.D., Hao X.G., Abudula A., Yoshida A., Guan G.Q. (2017). CuO nanowire@ Co_3_O_4_ ultrathin nanosheet core-shell arrays: An effective catalyst for oxygen evolution reaction. Electrochim. Acta.

[48] Woo S., Kim I., Lee J.K., Bong S., Lee J., Kim H. (2011). Preparation of cost-effective Pt–Co electrodes by pulse electrodeposition for PEMFC electrocatalysts. Electrochim. Acta.

[49] Aliofkhazraei M., Walsh F.C., Zangari G., Köçkar H., Alper M., Rizal C., Magagnin L., Protsenko V., Arunachalam R., Rezvanian A. (2021). Development of electrodeposited multilayer coatings: A review of fabrication, microstructure, properties and applications. Appl. Surf. Sci. Adv..

[50] Liu Y., Song Q.M., Zhang L.G., Xu Z.M. (2022). Targeted recovery of Ag-Pd alloy from polymetallic electronic waste leaching solution via green electrodeposition technology and its mechanism. Sep. Purif. Technol..

[51] Wang Q., Wang J., Jiang S., Li P. (2019). Recent Progress in Sol-Gel Method for Designing and Preparing Metallic and Alloy Nanocrystals. Acta Phys.-Chim. Sin..

[52] Jiang Y.W., Yang S.G., Hua Z.H., Huang H.B. (2009). Sol–gel autocombustion synthesis of metals and metal alloys. Angew. Chem..

[53] Hua Z.H., Cao Z.W., Deng Y., Jiang Y.W., Yang S.G. (2011). Sol–gel autocombustion synthesis of Co–Ni alloy powder. Mater. Chem. Phys..

[54] Bokov D., Jalil A.T., Chupradit S., Suksatan W., Ansari M.J., Shewael I.H., Valiev G.H., Kianfar E. (2021). Nanomaterial by sol-gel method: Synthesis and application. Adv. Mater. Sci. Eng..

[55] Wang Y.D., Jie S., Zhang G., Mu P.X., Wang X.Q., Li S.Y., Qiao L.L., Mu H.B. (2023). Advances in sol-gel-based superhydrophobic coatings for wood: A review. Int. J. Mol. Sci..

[56] Zhan J., Yue J., Zhang C., Fan Y. (2010). Research on preparation and application of quasi one-dimensional Ni-Co alloy materials. Mater. Guide.

[57] Tian M.B. (2005). Introduction to Materials Science.

[58] Yang X., Lu X.F., Zhang W., Guo X., Ren J.Q., Xue H.T., Tang F.L. (2023). Preparation and application of nano-Ni–Co alloy. J. Nanoparticle Res..

[59] Usami T., Salman S.A., Kuroda K., Gouda M.K., Mahdy A., Okido M. (2021). Synthesis of Cobalt-Nickel Nanoparticles via a Liquid-Phase Reduction Process. J. Nanotechnol..

[60] He N., He Z.D., Liu L., Lu Y., Wang F.Q., Wu W.H., Tong G.X. (2020). Ni^2+^ guided phase/structure evolution and ultra-wide bandwidth microwave absorption of Co_x_Ni_1−x_ alloy hollow microspheres. Chem. Eng. J..

[61] Eom H., Hwang I.-H., Lee D.Y., Lee S.M., Kim S.S. (2020). Preparation of liquid-phase reduction method-based Pt/TiO_2_ catalyst and reaction characteristics during HCHO room-temperature oxidation. Ind. Eng. Chem. Res..

[62] Dennis J.K., Jones D. (1981). Brush plating. Surf. Technol..

[63] Zheng W., Zhuang Z., Dai P., Zhao Y., Xie Y. (2010). Study on the microstructure and properties of nanocrystalline Ni Co alloy coating by electric brush plating. J. Fuzhou Univ. (Nat. Sci. Ed.).

[64] Fang X.Q., Xu H., Jin G.Q., Wang Y. (2017). Effect of magnetic field on brush plating Ni–Co alloy. Surf. Eng..

[65] Jin G., Ding X.L., Hu Z.F., Lv B., Wang X.H. (2019). Corrosion resistance of Ni graphene composite coating by electric brush plating. Rare Met. Mater. Eng..

[66] Wang X.H., Hu Z.F., Lv B., Yang Y.R., Liu X.B., Zhang H. (2019). Corrosion behavior of nickel cobalt nano alumina composite electric brush coating in NaCl solution. Electroplat. Finish..

[67] Suryanarayana C. (2001). Mechanical alloying and milling. Prog. Mater. Sci..

[68] Vaidya M., Muralikrishna G.M., Murty B.S. (2019). High-entropy alloys by mechanical alloying: A review. J. Mater. Res..

[69] Benjamin J.S. (1970). Dispersion strengthened superalloys by mechanical alloying. Metall. Trans..

[70] Olvera S., Sánchez-Marcos J., Palomares F.J., Salas E., Arce E.M., Herrasti P. (2014). Characterization and corrosion behaviour of CoNi alloys obtained by mechanical alloying. Mater. Charact..

[71] García-Contreras M.A., Fernández-Valverde S.M., Vargas-Garcia J.R. (2007). Oxygen reduction reaction on cobalt–nickel alloys prepared by mechanical alloying. J. Alloys Compd..

[72] Shuai C.J., He C.X., Peng S.P., Qi F.W., Wang G.Y., Min A.J., Yang W.J., Wang W.G. (2021). Mechanical alloying of immiscible metallic systems: Process, microstructure, and mechanism. Adv. Eng. Mater..

[73] Suryanarayana C. (2019). Mechanical alloying: A novel technique to synthesize advanced materials. Research.

[74] Burakowski T., Wierzchon T. (1998). Surface Engineering of Metals: Principles, Equipment, Technologies.

[75] Golodnitsky D., Rosenberg Y., Ulus A. (2002). The role of anion additives in the electrodeposition of nickel–cobalt alloys from sulfamate electrolyte. Electrochim. Acta.

[76] Benea L., Bonora P.L., Borello A., Martelli S. (2001). Wear corrosion properties of nano-structured SiC–nickel composite coatings obtained by electroplating. Wear.

[77] Baghery P., Farzam M., Mousavi A.B., Hosseini M. (2010). Ni–TiO_2_ nanocomposite coating with high resistance to corrosion and wear. Surf. Coat. Technol..

[78] Wang X., Zhou Z., Huang D. (2018). Research progress on the preparation of metal based particle thin films by composite electrodeposition technology. Hot Work. Technol..

[79] Tian Z.J., Wang D.S., Wang G.F., Shen L.D., Liu Z.D., Huang Y.H. (2010). Microstructure and properties of nanocrystalline nickel coatings prepared by pulse jet electrodeposition. Trans. Nonferrous Met. Soc. China.

[80] Low C.T.J., Wills R.G.A., Walsh F.C. (2006). Electrodeposition of composite coatings containing nanoparticles in a metal deposit. Surf. Coat. Technol..

[81] Borkar T., Harimkar S.P. (2011). Effect of electrodeposition conditions and reinforcement content on microstructure and tribological properties of nickel composite coatings. Surf. Coat. Technol..

[82] Kerr C., Barker D., Walsh F., Archer J. (2000). The electrodeposition of composite coatings based on metal matrix-included particle deposits. Trans. IMF.

[83] Benea L., Bonora P.L., Borello A., Martelli S., Wenger F., Ponthiaux P., Galland J. (2001). Composite electrodeposition to obtain nanostructured coatings. J. Electrochem. Soc..

[84] Zhang Y.C., Wan F.Q., Qi Q.X., Wang J.Y. (1995). Research on Electrodeposited Ni-Co Alloy. Plat. Finish..

[85] Wang D., Gao J.C., Zhang J.Q., Cheng Y.F., Jin H.M. (2013). Study on the Influence of Deposition Parameters on the Composition and Microstructure of Ni Co Coatings. J. Sichuan Univ. (Nat. Sci. Ed.).

[86] Chen Y.L., Yang H.S., Feng H., Yang P., Zhang J., Shu B.P. (2023). Electrodeposition and corrosion performance of Ni-Co alloys with different cobalt contents. Mater. Today Commun..

[87] Ling W., Wang H. (2023). Study on electrochemical properties of cobalt-nickel alloy prepared by pulsed electrodeposition. Int. J. Electrochem. Sci..

[88] Yao S.W., Liu B., Zhang W.G., Guo H.T., Shan X.X. (1996). Study on Electrodeposited Ni-Co Alloy and Its Structure. Plat. Finish..

[89] Wu G., Li N., Du M.H., Zhou D.R. (2002). Study on the Structure and Hardness of Electrodeposited Co Ni Alloy Coating. Mater. Sci. Technol..

[90] Wang L.P., Gao Y., Xue Q.J., Liu H.W., Xu T. (2005). Microstructure and tribological properties of electrodeposited Ni–Co alloy deposits. Appl. Surf. Sci..

[91] Wu Z.W., Lei Y.P., Wang Y., Fu H.G. (2013). Effect of cobalt content on microstructure and property of electroplated nickel-cobalt alloy coatings. Mater. Und Werkst..

[92] Hong S.H., Ahn S.H., Choi I., Pyo S.G., Kim H.J., Jang J.H., Kim S.K. (2014). Fabrication and evaluation of nickel cobalt alloy electrocatalysts for alkaline water splitting. Appl. Surf. Sci..

[93] Srivastava M., Selvi V.E., Grips V.K.W., Rajam K.S. (2006). Corrosion resistance and microstructure of electrodeposited nickel–cobalt alloy coatings. Surf. Coat. Technol..

[94] Zamani M., Amadeh A., Baghal S.M.L. (2016). Effect of Co content on electrodeposition mechanism and mechanical properties of electrodeposited Ni–Co alloy. Trans. Nonferrous Met. Soc. China.

[95] Karpuz A., Kockar H., Alper M., Karaagac O., Haciismailoglu M. (2012). Electrodeposited Ni–Co films from electrolytes with different Co contents. Appl. Surf. Sci..

[96] Tebbakh S., Mentar L., Messaoudi Y., Khelladi M.R., Belhadj H., Azizi A. (2021). Effect of cobalt content on electrodeposition and properties of Co–Ni alloy thin films. Inorg. Nano-Met. Chem..

[97] Hagarova M., Jakubéczyová D., Cervová J. (2015). Microstructure and properties of electroplated Ni-Co alloy coatings. Int. J. Electrochem. Sci..

[98] Tury B., Lakatos-Varsányi M., Roy S. (2006). Ni–Co alloys plated by pulse currents. Surf. Coat. Technol..

[99] Tury B., Lakatos-Varsányi M., Roy S. (2007). Effect of pulse parameters on the passive layer formation on pulse plated Ni–Co alloys. Appl. Surf. Sci..

[100] Chen Y.R., Long J.M., Pei H.Z., Hong J.P., Shi X.Z. (2009). Study on Internal Stress and Cobalt Content of Pulse Plated Ni Co Alloy Coating. Plat. Finish..

[101] Liu X.W., Xu Y.H., Hu P., Qu Y., Sun L.L. (2013). Wear resistance of high-frequency pulse electroplated nickel cobalt alloy coatings. Electroplat. Finish..

[102] Chen Y.L., Wen X.Y., Li H.G., Zhu F.K., Fang C., Li Z.Q., Zhou Z., Jiang W. (2023). Effects of deposition current density, time and scanning velocity on scanning jet electrodeposition of Ni-Co alloy coating. J. Manuf. Process..

[103] Li Y.D., Jiang H., Huang W.H., Tian H. (2008). Effects of peak current density on the mechanical properties of nanocrystalline Ni–Co alloys produced by pulse electrodeposition. Appl. Surf. Sci..

[104] Liu G.Z., Chen Z.L., Luo F., Liu T., Xi X.L., Wang Z.H., Gao Z., Shao P.H., Wu D.S., Luo X.B. (2023). One-step nickel-cobalt alloy electrodeposition from spent lithium-ion battery via synergistic pH adjustment and Mn^2+^ supplementation. Sep. Purif. Technol..

[105] Tian L., Xu J., Xiao S. (2011). The influence of pH and bath composition on the properties of Ni–Co coatings synthesized by electrodeposition. Vacuum.

[106] Vazquez-Arenas J., Altamirano-Garcia L., Treeratanaphitak T., Pritzker M., Luna-Sánchez R., Cabrera-Sierra R. (2012). Co–Ni alloy electrodeposition under different conditions of pH, current and composition. Electrochim. Acta.

[107] Liang Y., Xu Y.H., Li X., Zheng F.C., Zheng H.Q. (2010). Effect of process parameters on the corrosion resistance of high-frequency pulse electroplating nickel cobalt alloy in NaOH solution. Corros. Prot..

[108] Idris J., Christian C., Gaius E. (2013). Nanocrystalline Ni-Co Alloy Synthesis by High Speed Electrodeposition. J. Nanomater..

[109] Qiao G.Y., Jing T.F., Wang N., Gao Y.W., Zhao X., Zhou J.F., Wang W. (2005). High-speed jet electrodeposition and microstructure of nanocrystalline Ni–Co alloys. Electrochim. Acta.

[110] Zhang Y. (2015). Electrodeposited MCrAlY coatings for gas turbine engine applications. JOM.

[111] Huang L.F., Liu J.M., Wang S., Liu T., Shen J., Zhang D.M. (2019). Development Trends of Composite Electroplating Technology and Applications. Therm. Spray Technol..

[112] Walsh F.C., Larson C. (2020). Towards improved electroplating of metal-particle composite coatings. Trans. IMF.

[113] Walsh F.C., Wang S., Zhou N. (2020). The electrodeposition of composite coatings: Diversity, applications and challenges. Curr. Opin. Electrochem..

[114] Huang J.L., Wang Q.W., Yang Y.F., Wang X.M., Zhao Y., Zhu S., Li W. (2021). Research progress on silicon carbide composite electroplating technology as an alternative to chromium electroplating. Surf. Technol..

[115] Gao H., Bu L., Wang W. (2016). A Brief Discussion on the Development and Application of Electroplating Technology. Plat. Finish..

[116] Wang G.F., Jiang S.S., Lu Z., Zhang K.F. (2011). Preparation and tensile properties of Al_2_O_3_/Ni-Co nanocomposites. Trans. Nonferrous Met. Soc. China.

[117] Tian Y., Chengzhang P., Wei X. (2015). Ni Co Cr alloy co deposition process. J. Hunan Univ. Sci. Technol. (Nat. Sci. Ed.).

[118] Zhang Z., Li B.S., Chen S.Q., Yuan Z.W., Xu C.Y., Zhang W.W. (2024). Influences of Al particles and current density on structural, mechanical and anti-corrosion properties of electrodeposited Ni–Co/Al composite coatings. Ceram. Int..

[119] Li B., Zhang W., Li D. (2020). Synthesis and properties of a novel Ni–Co and Ni–Co/ZrO_2_ composite coating by DC electrodeposition. J. Alloys Compd..

[120] Wu G., Li N., Zhou D.R., Mitsuo K. (2004). Electrodeposited Co–Ni–Al_2_O_3_ composite coatings. Surf. Coat. Technol..

[121] Elkhoshkhany N., Hafnway A., Khaled A. (2017). Electrodeposition and corrosion behavior of nano-structured Ni-WC and Ni-Co-WC composite coating. J. Alloys Compd..

[122] Ma L., Zhou K.C., Li Z.Y., Wei Q.P. (2010). Electrodeposition of Ni-Co-Fe_2_O_3_ composite coatings. J. Cent. South Univ. Technol..

[123] Torabinejad V., Aliofkhazraei M., Rouhaghdam A.S., Allahyarzadeh M.H. (2017). Tribological properties of Ni-Fe-Co multilayer coatings fabricated by pulse electrodeposition. Tribol. Int..

[124] Kumaraguru S., Gnanamuthu R.M. (2021). An efficient corrosion protection activity of electrodeposited Ni/Ni-Co-Mn oxide composite for surface modification of steel. Mater. Lett..

[125] Apelt S., Zhang Y., Zhu J.H., Leyens C. (2015). Electrodeposition of Co–Mn_3_O_4_ composite coatings. Surf. Coat. Technol..

[126] Chang L.M., Guo H.F., An M.Z. (2008). Electrodeposition of Ni–Co/Al_2_O_3_ composite coating by pulse reverse method under ultrasonic condition. Mater. Lett..

[127] Shi L., Sun C., Liu W. (2008). Electrodeposited nickel–cobalt composite coating containing MoS_2_. Appl. Surf. Sci..

[128] Ghazanlou S.I., Farhood A.H.S., Ahmadiyeh S., Ziyaei E., Rasooli A., Hosseinpour S. (2019). Characterization of pulse and direct current methods for electrodeposition of Ni-Co composite coatings reinforced with nano and micro ZnO particles. Metall. Mater. Trans. A.

[129] Choudhary R.K., Mishra P., Kain V. (2017). Pulse DC electrodeposition of Zn–Ni–Co coatings. Surf. Eng..

[130] Arzumanova A.V., Starunov A.V., Shpanova K.A. (2019). Wear Resistance of a Composite Galvanic Coating Based on the Nickel-Cobalt Alloy. Mater. Sci. Forum.

[131] Wang L.P., Gao Y., Xue Q.J., Liu H.W., Xu T. (2005). Study on the Wear Resistance of Ni Co/Nanodiamond Composite Coating. China Surface Eng..

[132] Xu W., Xu T., Wang X.L., Wang L.L., Tan Y.F. (2015). Effect of Diamond Particle Size on the Microstructure and Wear Resistance of Ni Co/Diamond Composite Coating. Equip. Manuf. Technol..

[133] Shi L., Sun C.F., Gao P., Zhou F., Liu W.M. (2006). Electrodeposition and characterization of Ni–Co–carbon nanotubes composite coatings. Surf. Coat. Technol..

[134] Shi L., Sun C.F., Zhou F., Liu W.M. (2005). Electrodeposited nickel–cobalt composite coating containing nano-sized Si_3_N_4_. Mater. Sci. Eng. A.

[135] Ghazanlou S.I., Farhood A.H.S., Hosouli S., Ahmadiyeh S., Rasooli A. (2018). Pulse and direct electrodeposition of Ni–Co/micro and nanosized SiO_2_ particles. Mater. Manuf. Process..

[136] Ghazanlou S.I., Ahmadiyeh S., Yavari R. (2017). Investigation of pulse electrodeposited Ni–Co/SiO_2_ nanocomposite coating. Surf. Eng..

[137] Ghazanlou S.I., Shokuhfar A., Navazani S., Yavari R. (2016). Influence of pulse electrodeposition parameters on microhardness, grain size and surface morphology of Ni–Co/SiO_2_ nanocomposite coating. Bull. Mater. Sci..

[138] Akbarpour M.R., Asl F.G., Rashedi H. (2023). Pulse-reverse electrodeposition of Ni-Co/graphene composite films with high hardness and electrochemical behaviour. Diam. Relat. Mater..

[139] Ramesh C.S., Seshadri S.K. (2003). Tribological characteristics of nickel based composite coatings. Wear.

[140] Garcia I., Fransaer J., Celis J.P. (2001). Electrodeposition and sliding wear resistance of nickel composite coatings containing micron and submicron SiC particles. Surf. Coat. Technol..

[141] Benea L., Bonora P.L., Borello A., Martelli S. (2002). Effect of SiC size dimensions on the corrosion wear resistance of the electrodeposited composite coating. Mater. Corros..

[142] Carter C.H., Davis R.F., Bentley J. (1984). Kinetics and mechanisms of high-temperature creep in silicon carbide: II, chemically vapor deposited. J. Am. Ceram. Soc..

[143] Gulbransen E.A., Jansson S.A. (1972). The high-temperature oxidation, reduction, and volatilization reactions of silicon and silicon carbide. Oxid. Met..

[144] Price R.J. (1977). Properties of silicon carbide for nuclear fuel particle coatings. Nucl. Technol..

[145] Snead L.L., Nozawa T., Katoh Y., Byun T.S., Kondoa S., Petti D.A. (2007). Handbook of SiC properties for fuel performance modeling. J. Nucl. Mater..

[146] Bakhit B., Akbari A., Nasirpouri F., Hosseini M.G. (2014). Corrosion resistance of Ni–Co alloy and Ni–Co/SiC nanocomposite coatings electrodeposited by sediment codeposition technique. Appl. Surf. Sci..

[147] Dai P.Q., Zhong Y.H., Zhou X. (2011). Corrosion characteristic of pulsed electrodeposition Ni–Co/SiC nanocomposite coating. Surf. Eng..

[148] Shi L., Sun C., Gao P., Zhou F., Liu W. (2006). Mechanical properties and wear and corrosion resistance of electrodeposited Ni–Co/SiC nanocomposite coating. Appl. Surf. Sci..

[149] Srivastava M., William Grips V.K., Jain A., Rajam K.S. (2007). Influence of SiC particle size on the structure and tribological properties of Ni–Co composites. Surf. Coat. Technol..

[150] Shi L., Zhou F., Sun C.F., Liu W.M. (2005). Corrosion resistance and tribological properties of Ni Co SiC nanocomposite coating. Chin. J. Nonferrous Met..

[151] Kamel M.M., Mohsen Q., Abdel Hamid Z., Rashwan S.M., Ibrahim I.S., El-Sheikh S.M. (2021). Electrodeposition of Ni-Co/Nano SiC Composites from a Citrate Bath and their Characterization. Int. J. Electrochem. Sci..

[152] Ababsa A., Ben Temam H., Hasan G.G., Althamthami M., Malfi A.N. (2022). Effect of sodium dodecyl sulfate and different SiC quantities on electrodeposited Ni-Co alloy coatings. Surface Topogr. Metrol. Prop..

[153] Yang Y., Cheng Y.F. (2013). Mechanistic aspects of electrodeposition of Ni–Co–SiC composite nano-coating on carbon steel. Electrochim. Acta.

[154] Bahadormanesh B., Dolati A. (2010). The kinetics of Ni–Co/SiC composite coatings electrodeposition. J. Alloys Compd..

